# Dose-Dependent Activation of Putative Oncogene *SBSN* by BORIS

**DOI:** 10.1371/journal.pone.0040389

**Published:** 2012-07-05

**Authors:** Daria Gaykalova, Rajita Vatapalli, Chad A. Glazer, Sheetal Bhan, Chunbo Shao, David Sidransky, Patrick K. Ha, Joseph A. Califano

**Affiliations:** 1 Department of Otolaryngology–Head and Neck Surgery, Johns Hopkins Medical Institutions, Baltimore, Maryland, United States of America; 2 Department of Oncology, Johns Hopkins Medical Institutions, Baltimore, Maryland, United States of America; 3 Milton J. Dance Head and Neck Center, Greater Baltimore Medical Center, Baltimore, Maryland, United States of America; University of Bristol, United Kingdom

## Abstract

Testis-specific transcription factor BORIS (Brother of the Regulator of Imprinted Sites), a paralog and proposed functional antagonist of the widely expressed CTCF, is abnormally expressed in multiple tumor types and has been implicated in the epigenetic activation of cancer-testis antigens (CTAs). We have reported previously that suprabasin (*SBSN*), whose expression is restricted to the epidermis, is epigenetically derepressed in lung cancer. In this work, we establish that *SBSN* is a novel non-CTA target of BORIS epigenetic regulation. With the use of a doxycycline-inducible BORIS expressing vector, we demonstrate that relative BORIS dosage is critical for *SBSN* activation. At lower concentrations, BORIS induces demethylation of the *SBSN* CpG island and disruption and activation of chromatin around the *SBSN* transcription start site (TSS), resulting in a 35-fold increase in *SBSN* expression in the H358 human lung cancer cell line. Interestingly, increasing BORIS concentrations leads to a subsequent reduction in *SBSN* expression via chromatin repression. In a similar manner, increase in BORIS concentrations leads to eventual decrease of cell growth and colony formation. This is the first report demonstrating that different amount of BORIS defines its varied effects on the expression of a target gene via chromatin structure reorganization.

## Introduction

BORIS (Brother of the Regulator of Imprinted Sites) is an 11 zinc finger, male germ line-specific transcription factor [1], [2]. Unlike its widely expressed paralog CTCF, BORIS is repressed in normal tissues and expressed only in the testis and in several types of human cancers, including lung, head and neck, breast, skin, and urinary cancers. It is therefore considered a cancer-testis antigen (CTA) [2], [3], [4], [5]. BORIS has been implicated in the activation of other CTAs such as *MAGEA1-A4* and *NY-ESO-1* [3], [6], [7]. BORIS was shown to play a role in promoter demethylation of *MAGEA1* and *MAGEA3* genes [6], [7]. The role of BORIS in chromatin activation has also been demonstrated for *MAGEA1-A4* and *BAG-1* genes [7], [8]. While the role of BORIS in the regulation of gene expression was demonstrated primarily for CTAs [9], [10], [11], [12], [13], its role on non-CTA genes is limited. Recent reports suggest that BORIS may bind and affect expression of *MYC, BRCA1, OCT4, hTERT*, and *Rb2/p130*, as well as the *H19* imprinting control region [4], [14], [15], [16]. We propose that BORIS may play a role in the regulation of another non-CTA oncogenic gene, *SBSN* [9].

Although we have demonstrated that BORIS can induce cell proliferation [10], the role of BORIS in carcinogenesis is controversial. BORIS has been demonstrated to have a diverse effect on the expression of its targets, similar to transcription factors CTCF and Sp1 [17], [18], [19]. BORIS can participate in both DNA methylation and demethylation of its target genes [3], [6], [7], [20]. BORIS-specific activation of *MAGEA2* and *MAGEA4* expression is independent of DNA methylation [7]. However, recent reports argue that BORIS is neither necessary nor sufficient for the activation of *MAGEA1* [21], [22] or for the development of breast cancer [23].

Abnormal *BORIS* induction varies by 10 to 10 000 000-fold among different cell types [2], [3], [5]. This variability in *BORIS* induction in different primary cancer types and cancer cell lines has been shown to be regulated by DNA methylation and by CTCF and p53 factors [24]. It has also been shown to be induced by the demethylating agent 5 Aza 2′-deoxycytidine (DAC) [3]. *BORIS* expression can also be activated through the chromosomal amplification of its locus, 20q13 [2]. This locus contains other known oncogenes, such as *AURKA, BCAS1, EEF1A2* and *GNAS* [2]. 20q13 has been shown to be amplified in multiple tumor types, including gastric, breast and lung cancers, and has often been associated with metastasis and poor prognosis [2], [25], [26]. BORIS induces aberrant cell growth of both normal human keratinocytes and normal mice embryonic cell lines [10], implicating a role for BORIS in tumorigenesis. Anti-BORIS vaccination leads to reduction of cancer growth in 20% of cases and a decrease in spontaneous metastasis in 50% of cases [27], [28]. In contrast, treatment of cancer with DAC, which generally stimulates a cancer-specific immune response [29], can result in unpredicted induction of BORIS by 10 to 1000-fold [3] and subsequent BORIS-mediated carcinogenesis. Therefore, BORIS may have different effects, depending on its concentration and the contextual setting of expression.

We have demonstrated earlier that the non-CTA gene suprabasin (*SBSN*), which is normally expressed only in the suprabasal layer of epidermis [30], [31], [32], was specifically demethylated at its CpG island and subsequently expressed in lung cancer tissues [9]. Abnormal upregulation of *SBSN* expression has also been demonstrated for glioblastoma [33]. We have demonstrated strong correlation of *BORIS* and *SBSN* expression in 190 lung cancer specimens [9]. We have also determined that SBSN induces cell proliferation in normal and lung cancer cell lines, and we propose that BORIS participates in aberrant *SBSN* expression in lung cancer [9].

In this work, we investigated the epigenetic regulation of the non-CTA gene *SBSN* by BORIS. We employed a doxycycline-inducible system to detail the dose-dependent effects of epigenetic regulation by BORIS on *SBSN* expression and on cell growth. This study demonstrates that different BORIS concentrations have different effects on target gene expression and on the cell proliferative response to *BORIS* expression.

## Materials and Methods

### Histopathology

This study was approved by the Johns Hopkins Institutional Review Board; all tissues were acquired under protocol NA_00001911. Written informed consent was obtained from each subject prior to the use of their tissue for scientific research. Samples were analyzed by the Pathology department at the Johns Hopkins Hospital. Tumor and normal lung tissues from surgical specimens were frozen in liquid nitrogen immediately after surgical resection and stored in liquid nitrogen until use. Tumor samples were confirmed to be non-small cell lung cancer (NSCLC) with at least 80% tumor purity. Normal samples were obtained from normal lung parenchyma. Tissue RNA extraction and qRT-PCR were performed as described below.

### Cell lines, Plasmids, Transfection

The lung cancer cell line H358 was obtained from American Type Culture Collection (ATCC, Manassas, VA, USA). Cells were cultured in RPMI1640 medium supplemented with 10% FBS and 1% penicillin/streptomycin and incubated in 37°C and 5% CO_2_. For ectopic BORIS expression, a BORIS expression plasmid (pBIG2i-BORIS, aka BORIS) and a control empty vector (EV) were used [6], [7]. H358 cells grown in 6-well plates were transfected with 2 µg of plasmid using Fugene HD (Roche, Indianapolis, IN, USA). Twenty-four hours post-transfection, cells were induced with 0, 0.0313 or 1 µg/ml final concentrations of doxycycline in growth media and were allowed to grow for 48 hours before harvesting for RNA or DNA extraction. For ChIP and nucleosome occupancy analyses, cells were grown in 150 cm^2^ dishes and transfected with 30 µg of the BORIS expression plasmid or the control empty vector. Cells were then induced with doxycycline as described above and harvested. In all experiments, transfection efficiency was evaluated by GFP fluorescence of cells transfected in parallel with the experiment with an equal amount of pCMV6-AC-GFP (Origene, Rockville, MD, USA). Overall transfection efficiency was at least 70%.

### Cell proliferation assay

H358 cells were seeded in 96-well plates and allowed to grow in RPMI1640 medium until the cells were approximately 70% confluent. Cells were transfected with a BORIS expressing vector or control empty vector and induced by 0, 0.0313 or 1 µg/ml doxycycline 24 hours post-transfection. Cell metabolic activity was determined every 24 hours using the CCK-8 colorimetric assay (Dojindo, Gaitherburg, MD, USA) at 450 nm according to the manufacturer's instructions. Values are mean ± SEM for pentaplicates of cultured cells. P-values were evaluated for differences between empty vector and BORIS inductions.

### RNA extraction, Reverse transcription and quantitative real-time PCR (qRT-PCR)

Total RNA was extracted using Trizol (Life Technologies, Gaithersburg, MD, USA) and the RNeasy Plus Kit (Qiagen, Valencia, CA, USA) according to the manufacturers' instructions. 1 µg of RNA was reverse transcribed using the High Capacity cDNA Reverse Transcription Kit (Applied Biosystems, Carlsbad, CA, USA). Quantitative real-time PCR was performed using gene-specific Applied Biosystems-recommended expression assays (Table S1) and Universal PCR Master mix (both from Applied Biosystems) on the 7900HT real time PCR machine (Applied Biosystems). Expression of the gene of interest was quantified relative to GAPDH expression.

### DNA extraction and Bisulfite treatment

For DNA extraction, cells were harvested and incubated in 10% SDS supplemented with 1% proteinase K for 48 hours. DNA was extracted with phenol-chloroform and resuspended in LoTE buffer (EDTA 2.5 mM and Tris-HCl 10 mM, pH 7.5). 2 µg of DNA was bisulfite converted and purified using the EpiTect Bisulfite Kit (Qiagen, Valencia, CA, USA) according to the manufacturer's instructions and stored at −80°C.

### Quantitative methylation-specific PCR (QMSP)

Bisulfite-converted DNA was used for QMSP as previously described [7]. Real-time PCR was performed using Universal PCR Master Mix (Applied Biosystems) on the 7900HT real-time PCR machine with normalization to unmethylated beta-actin control [34]. Sequences of the primers and probes used can be found in Table S1.

### Chromatin-Immunoprecipitation assay (ChIP) and quantitative real-time PCR (qRT-PCR)

BORIS-transfected H358 cells after 48 hours of doxycycline treatment were used for ChIP assay, performed using the Magna ChIP™ G Chromatin Immunoprecipitation Kit (Millipore, Billerica, MA, USA) according to the manufacturer's instructions. Anti-BORIS antibodies were from Abcam (Cambridge, MA, USA); all other antibodies were from Millipore. Non-specific rabbit IgG antibodies were used as controls for quantification. DNA concentration was measured, and DNA was stored at −80°C. Equal amounts of DNA from each sample was used for qRT-PCR with specific primers (Table S1) and SYBR Green Master Mix (Applied Biosystems) on the 7900HT real-time PCR machine with normalization to rabbit IgG antibodies for quantification.

### Nucleosome Occupancy using primer Extension

To map the nucleosome occupancy around the transcription start site of *SBSN*, chromatin isolation and primer extension protocols were used. In short, BORIS-transfected H358 cells after 48 hours of doxycycline treatment were SDS-lysed, and the chromatin was treated with 10 U/µl final concentration of MNase for 0 to 60 minutes as previously described [35]. DNA was extracted with phenol-chloroform and resuspended in LoTE. DNA from a single time point, 8 min in this work, was used for primer extension with a standard PCR program with a single 5′-biotin primer designed to span the promoter regions of *SBSN*. Products were resolved using 6M Urea 5% 19:1 PAGE. Gel-resolved PCR products were transferred to a positively charged Hybond N+ nylon membrane (Amersham Pharmacia Biotech, Cleveland, OH, USA) by blotting and detected using the Chemiluminescent Nucleic Acid Detection kit (Thermo Scientific, Rockford, IL, USA) per the manufacturer's instructions. The membrane was exposed to X-ray film (Kodak, New York, NY, USA) and developed to visualize the products.

## Results

### 1. Expression of *SBSN* and *BORIS* are directly correlated

**Figure 1:**
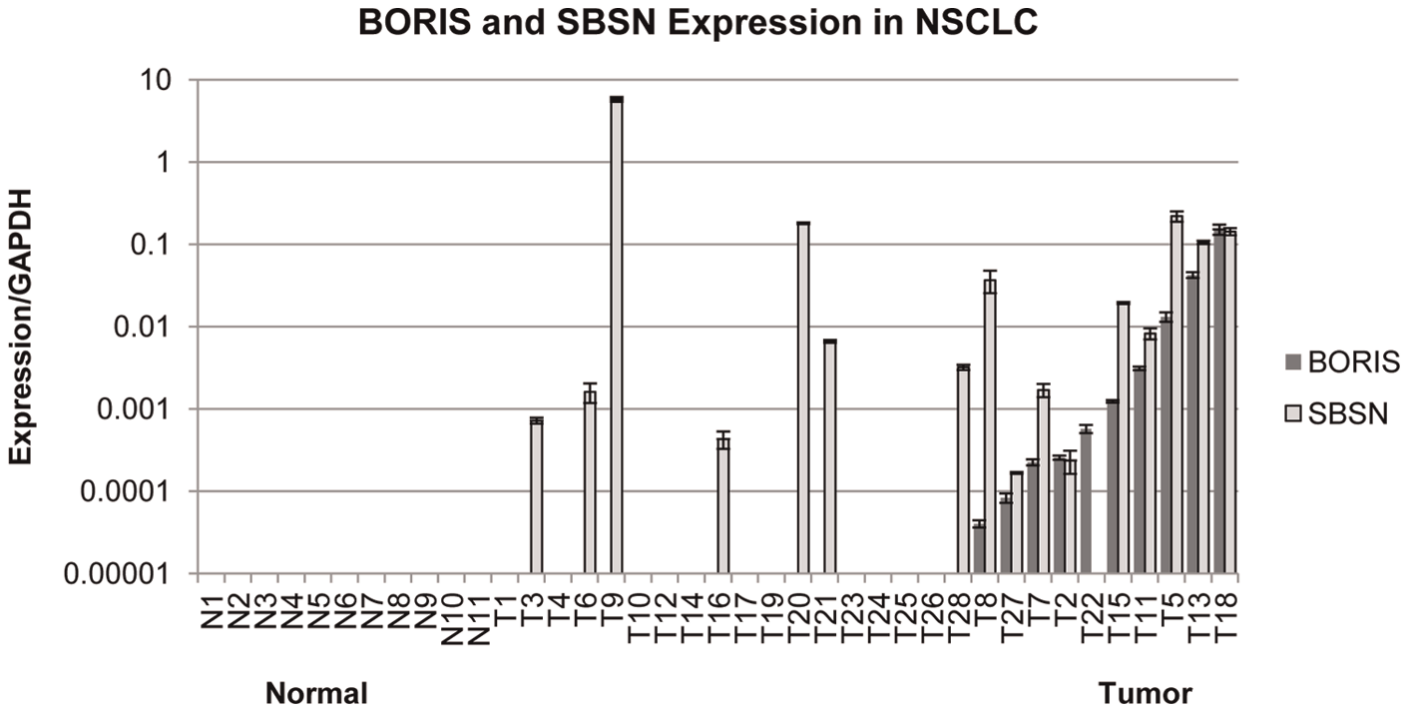
*BORIS* and *SBSN* expression in NSCLC clinical samples. *BORIS* and *SBSN* expression in 11 samples from healthy individuals (N, left) and 28 samples from NSCLC patients (T, right). Expression was quantified relative to GAPDH. *SBSN* and *BORIS* co-express with p-value  = 0.0004 (by Fisher exact test). Clinical samples were ranked by *BORIS* expression level.

We have demonstrated in 190 primary lung cancer samples that *SBSN* is overexpressed in lung cancer and propose that *SBSN* expression is dependent on *BORIS* [9]. To evaluate the BORIS-dependent expression of *SBSN*, we checked expression of both genes on a smaller cohort of 11 normal and 28 non-small cell lung cancer samples (Figure 1). The expression of both genes were strongly correlated (p = 0.0004). These results suggest that BORIS, as a transcription factor, is involved in the transcriptional regulation of *SBSN* expression. Protein levels of BORIS and SBSN could not be validated in primary tissues, due to the unavailability of appropriate antibodies for Western blot or immunohistochemistry staining for both BORIS and SBSN proteins.

### 2. BORIS directly binds to the *SBSN* gene

**Figure 2:**
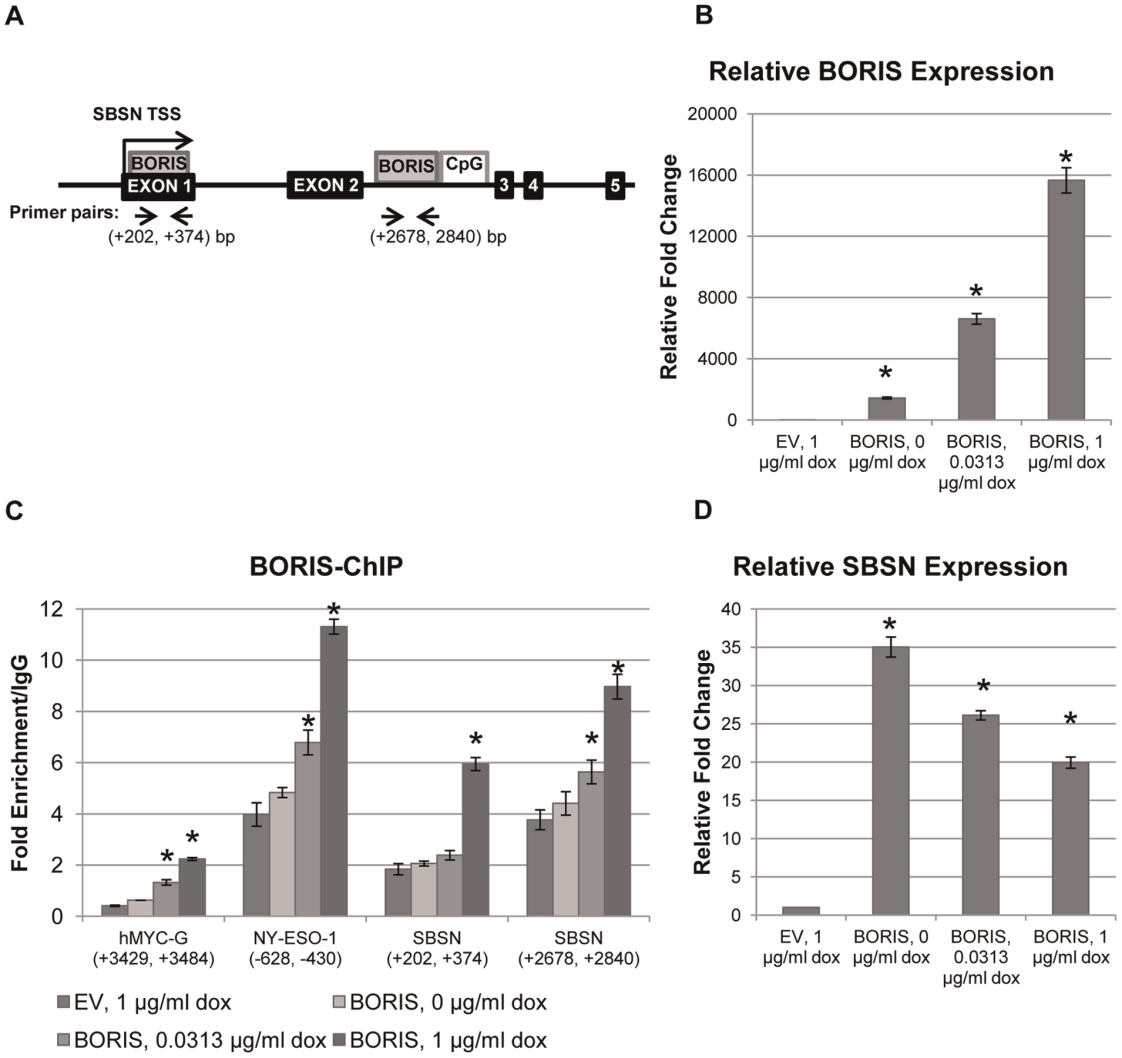
*SBSN* is a target of dose-dependent BORIS transcriptional regulation. (A) Schematic structure of the *SBSN* gene. The relative positions of exons, CpG islands, and CTCF/BORIS (BORIS) binding sites are shown in black, white and grey, respectively. Transcription start sites (TSS) at the +1 position are indicated by arrows. TSS-relative positions of primers used in qRT-PCR reactions from ChIP-purified DNA are indicated by arrow heads. (B) Relative BORIS mRNA level in the H358 cell line after transient transfection of BORIS. BORIS expression was induced by indicated concentrations of doxycycline (dox) 24 hours after transfection with control empty vector (EV) or with BORIS expressing vector. Expression was quantified relative to GAPDH with the control referred as 1 (*, p-value <0.00005 (*t* test)). (C) BORIS binding at two prospective BORIS binding sites at *SBSN*, positive control *NY-ESO-1*, and at negative control *hMYC-G* non-binding site [24], shown in (A), as analyzed by qRT-PCR from ChIP DNA. Fold enrichment is relative to IgG binding (*, p-value <0.04; here and further below unlabeled bars are p-value >0.05). (D) Expression of *SBSN* after transient transfection of BORIS. cDNA from the experiment in (B) was used for qRT-PCR. *SBSN* expression was quantified relative to *GAPDH* expression and normalized to control referred as 1 (*, p-value <0.00006). Mean value ± SEM for 3 replicates is shown for all experiments.

To test the hypotheses that BORIS stimulates *SBSN* expression and that *SBSN* is a direct target of BORIS, we looked for possible CTCF/BORIS binding sites within the *SBSN* promoter and coding regions by sequence homology to previously published CTCF/BORIS binding sites [2], [3], [19]. We identified two prospective CTCF/BORIS binding sites within the *SBSN* gene sequence (Figure 2A). The first, which had greater homology to known CTCF/BORIS binding sites, was downstream to the *SBSN* transcription start site (TSS), in the second intron in close proximity to the *SBSN* CpG island. The second, an upstream binding site, was found in the first exon next to the *SBSN* TSS.

To evaluate the effect of varying BORIS concentrations on the regulation of *SBSN* expression, we employed a doxycycline-inducible pBIG2i-BORIS expression plasmid for drug dose-dependant *BORIS* expression [6]. The bronchoalveolar p53-deficient non-small cell lung cancer (NSCLC) H358 cell line expresses relatively small amounts of BORIS [3] and was used for these *BORIS* induction experiments. Varied *BORIS* expression levels were induced by different doxycycline concentrations, ranging from 0 to 1 µg/ml. The use of tetracycline-regulated minimal CMV promoter of pBIG2i allows us to induce *BORIS* to sub-physiological BORIS concentrations found in clinical samples from NSCLC and other cancer types (compare Figure 1 and Figure S1, and [5]). There was basal BORIS expression in doxycycline-free media due to a leaky promoter in the vector; however, BORIS transcript was increased by 1 500 to 15 000-fold in H358 cells using doxycycline concentrations ranging from 0 to 1 µg/ml, respectively, compared to cells transfected with empty vector (EV) (Figure 2B).

The presence of BORIS at the proposed binding sites was evaluated by chromatin immunoprecipitation (ChIP) assay with BORIS-specific antibodies (Figure 2C). BORIS was strongly enriched at both BORIS/CTCF binding sites of *SBSN* compared to *c-MYC* non-binding site G, which served as a negative control [24]. BORIS was enriched at the downstream site 1.5-fold more than at the upstream site (Figure 2C). The presence of BORIS at these sites increased with increasing levels of its expression (Figures 2B and 2C). Note that BORIS occupancy at the *NY-ESO-1* promoter was used as a positive control, whereas Hong and colleagues demonstrated BORIS binding with *NY-ESO-1* DNA [3]. We found that BORIS binding to the newly discovered downstream binding site at *SBSN* is as strong as at the control *NY*-*ESO-1* gene site, achieving 9-fold enrichment at *SBSN* and 11-fold enrichment at *NY*-*ESO-1* (Figure 2C, 1 µg/ml doxycycline).

These data suggest that *SBSN* is a new BORIS target gene, in which BORIS binds with greater affinity to the downstream binding site adjacent to the CpG island.

### 3. BORIS dose-dependent transcriptional activation of *SBSN*

To evaluate the effect of BORIS on *SBSN* expression we measured *SBSN* mRNA levels in H358 after BORIS induction (Figure 2D). In the presence of lower BORIS concentrations at 0 µg/ml doxycycline, *SBSN* expression was increased by 35-fold compared to control. Surprisingly, at higher BORIS concentrations induced by 1 µg/ml doxycycline, *SBSN* expression was increased only by 20 times, half that induced by the lower BORIS concentrations.

To eliminate cell line- and organ-specific effects of different BORIS concentrations, the data were confirmed on A549 lung adenocarcinoma and HeLa cervical adenocarcinoma cell lines (Figure S2). Lower BORIS concentrations (0 µg/ml doxycycline) induced 3–4 fold *SBSN* expression in both cell lines, while increased BORIS concentrations (1 µg/ml doxycycline) led to a relative decrease in *SBSN* expression by 2–3 fold (Figure S2), resulting in only 1.5-fold activation in *SBSN* gene expression for both cell lines, as compared to controls.

Given these findings, we were interested to see the effect of BORIS knock-down on *SBSN* expression in a BORIS-positive cell line. For this reason, we employed the p53-deficient H1299 cell line, which has high baseline BORIS expression (Figure S3; [3]). We observed 5-fold reduction of *SBSN* expression after 35% BORIS knock-down (Figure S4).

Collectively, these data indicate that *SBSN* gene expression requires the presence of BORIS for its induction. However, different BORIS concentrations induce *SBSN* expression to a variable extent. Decreased BORIS concentrations by knock-down causes a reduction in *SBSN* expression, whereas high levels of BORIS expression cause an incremental decrease in overall *SBSN* expression relative to lower levels of induced BORIS expression.

### 4. BORIS induction of *SBSN* is associated with methylation of the *SBSN* CpG island

**Figure 3:**
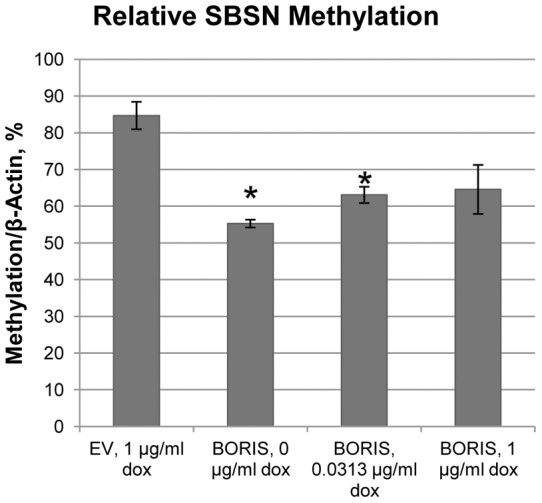
BORIS-dependent DNA demethylation of *SBSN* CpG island. DNA was isolated from H358 cells transfected with BORIS expressing vector and induced by indicated doxycycline concentrations. DNA was treated by bisulfite, purified and used for QMSP with *SBSN*-specific primers and probes at *SBSN* as described in methods. Values are normalized to beta-actin unmethylated control. (*, p-value <0.03).

The published data suggest that BORIS regulates expression of its targets via DNA methylation [3], [6], [7]. To evaluate the role of BORIS in the epigenetic regulation of *SBSN* gene expression, we analyzed the level of DNA methylation of the *SBSN* CpG island, located in the second intron of *SBSN* (Figure 2A). Quantitative methylation specific PCR (QMSP) revealed that induction of smaller BORIS concentrations in the H358 cell line leads to a large decrease in *SBSN* DNA methylation (Figure 3). Interestingly, gradual increases in BORIS concentration resulted in relative increases in *SBSN* DNA methylation, in agreement with the gradual decrease of *SBSN* induction (Figure 2D).

### 5. BORIS induction of *SBSN* expression results in chromatin structural reorganization

**Figure 4:**
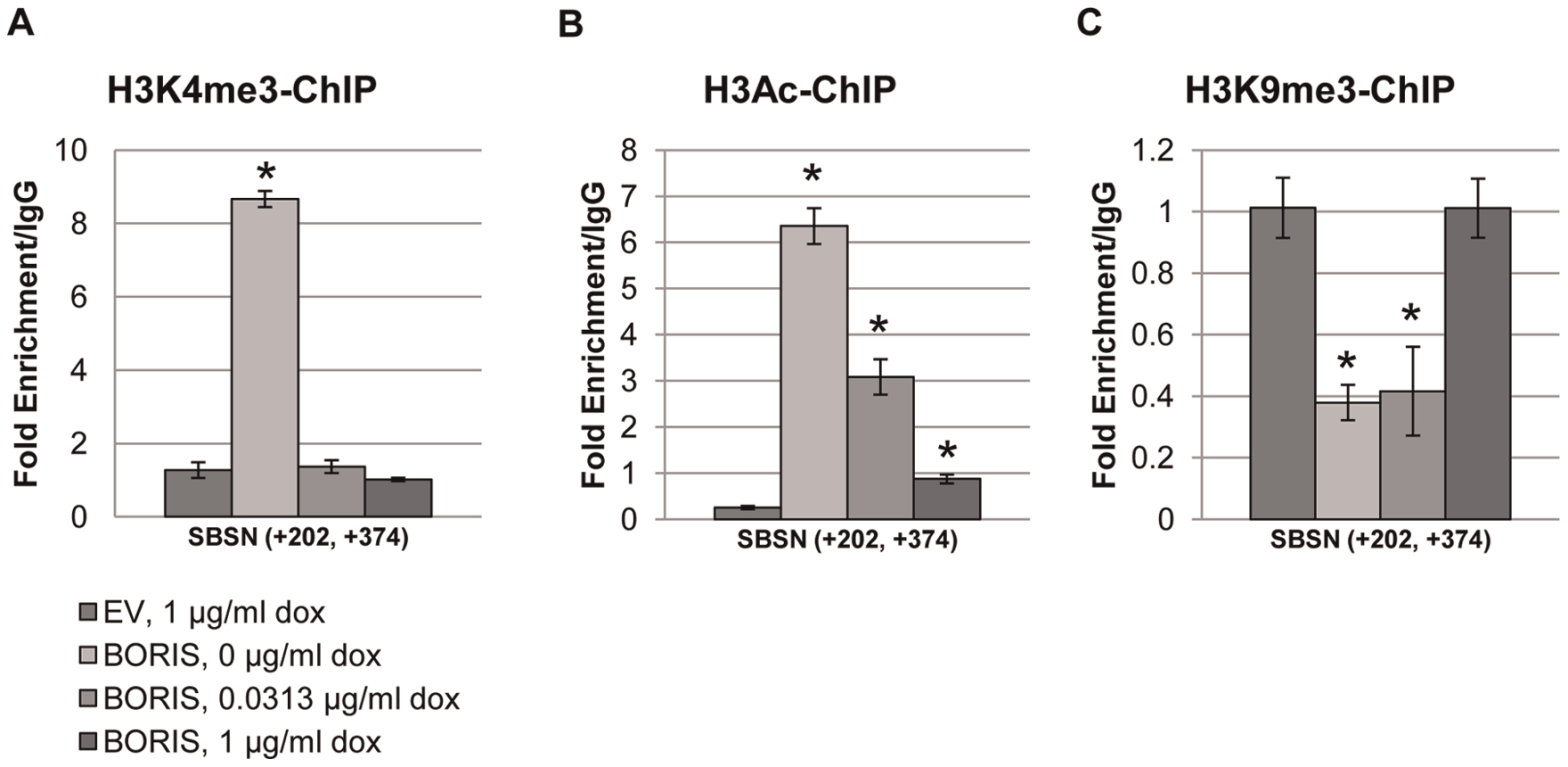
Changes in histone modifications around *SBSN* TSS upon BORIS induction. (A) Enrichment of active chromatin modification, H3K4me3, near the *SBSN* TSS was measured by ChIP experiment with H3K4me3 antibodies for H358 transfected with BORIS and induced by indicated doxycycline concentrations. The region from +202 bp to +374 bp was analyzed. Note that cell lysates from the same experiments was used for all ChIP experiments (Figure 2C). Enrichment level was measured relative to IgG in qRT-PCR as described in the Methods. (B) Enrichment of active chromatin modification – H3Ac. (C) Enrichment of repressive chromatin modification, H3K9me3. (*, p-value <0.00002 (A); p-value <0.004 (B); p-value <0.03 (C)).

BORIS is implicated in the chromatin activation of *MAGEA1-A4, BAG1, BRCA1* and *MYC* genes via the recruitment of active histone modifiers [3], [7], [8], [15]. We evaluated the possibility that *SBSN* expression was altered by chromatin structure modifications in a BORIS-dependent manner. Active histone modifications, trimethylation of lysine 4 of histone H3 tail (H3K4me3) [36], acetylation of lysine 14 of histone H3 tail (H3Ac) [37], and repressive trimethylation of lysineK9 of H3 (H3K9me3) [38], [39] of the *SBSN* gene were evaluated at different BORIS concentrations (Figure 4). Histone modifications of both H3K4me3 and H3Ac were enriched by 7- and 9–fold, respectively, at lowest BORIS concentrations and strongly correlate with an increase in *SBSN* expression (Figure 2D). Both modifications gradually decreased with increased BORIS concentration, induced by 1 µg/ml doxycycline (Figures 4A and B). In contrast, the repressive modification H3K9me3 was depleted 3-fold at lower BORIS concentrations and gradually increased to control levels at higher BORIS concentrations (Figure 4C). We confirmed the changes in chromatin structure induced by BORIS differential expression for the control gene *NY-ESO-1* and demonstrated similar kinetics of alterations in histone modifications with different BORIS concentrations (Figures S5A and C).

**Figure 5:**
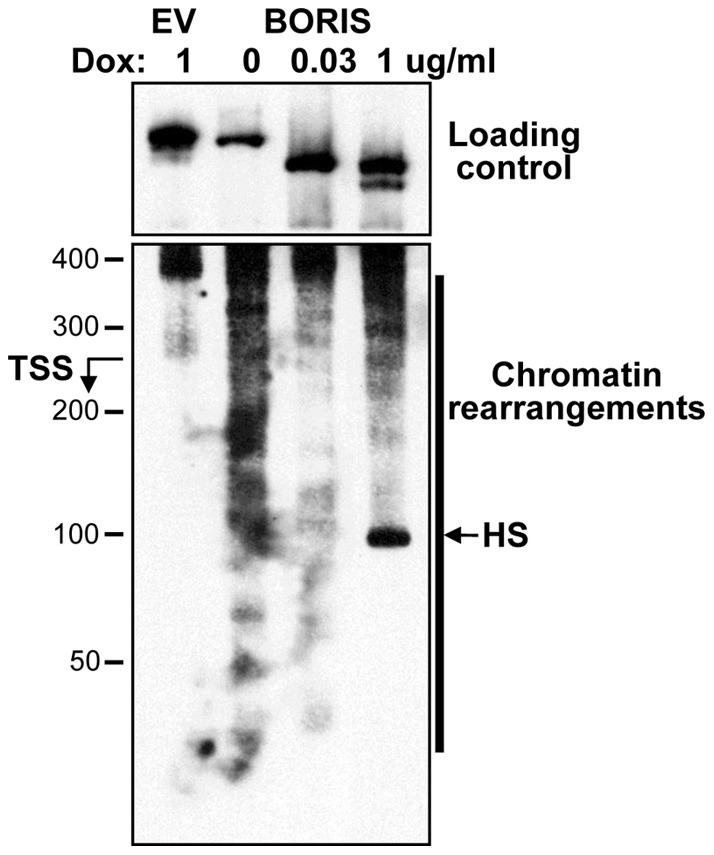
Reorganization of *SBSN* chromatin structure upon BORIS induction. *BORIS* expression was induced by 0, 0.0313 or 1 µg/ml doxycycline 24 hours after transfection with control empty vector or with BORIS expressing vector. Cell nuclei were isolated and digested with MNase. Purified DNA after 8 min of digestion with MNase (Figure S6) was used in PCR for primer extension experiments as described in the Methods. Relative localization of the TSS and region of chromatin reorganization is indicated. DNA digestion outside of *SBSN* promoter was used as loading control. Note: DNA was most accessible for MNase digestion at 0 µg/ml doxycycline, even at overall underloading of this sample. M – Hi-Lo DNA ladder (BioRad).

We also evaluated the overall nucleosome occupancy near the *SBSN* TSS, in the area of critical −1 and +1 nucleosomes (Figure 5). We have demonstrated strong chromatin opening at lower BORIS concentrations (0 µg/ml doxycycline) as compared to control, determined by greater DNA accessibility for digestion by micrococcal nuclease (MNase) (Figure S6). We also noticed decreased DNA accessibility with an increase in BORIS concentrations (Figure 5). We noted partial disruption of *SBSN* chromatin induced by BORIS at 1 µg/ml doxycycline (Figure 5); however, even under these conditions, chromatin is more condensed than at 0 µg/ml doxycycline.

Collectively, these data suggest that the chromatin structure of BORIS-bound *SBSN* is a primary target of BORIS epigenetic regulation.

### 6. Cell growth stimulatory effects of BORIS are dose-dependent

**Figure 6:**
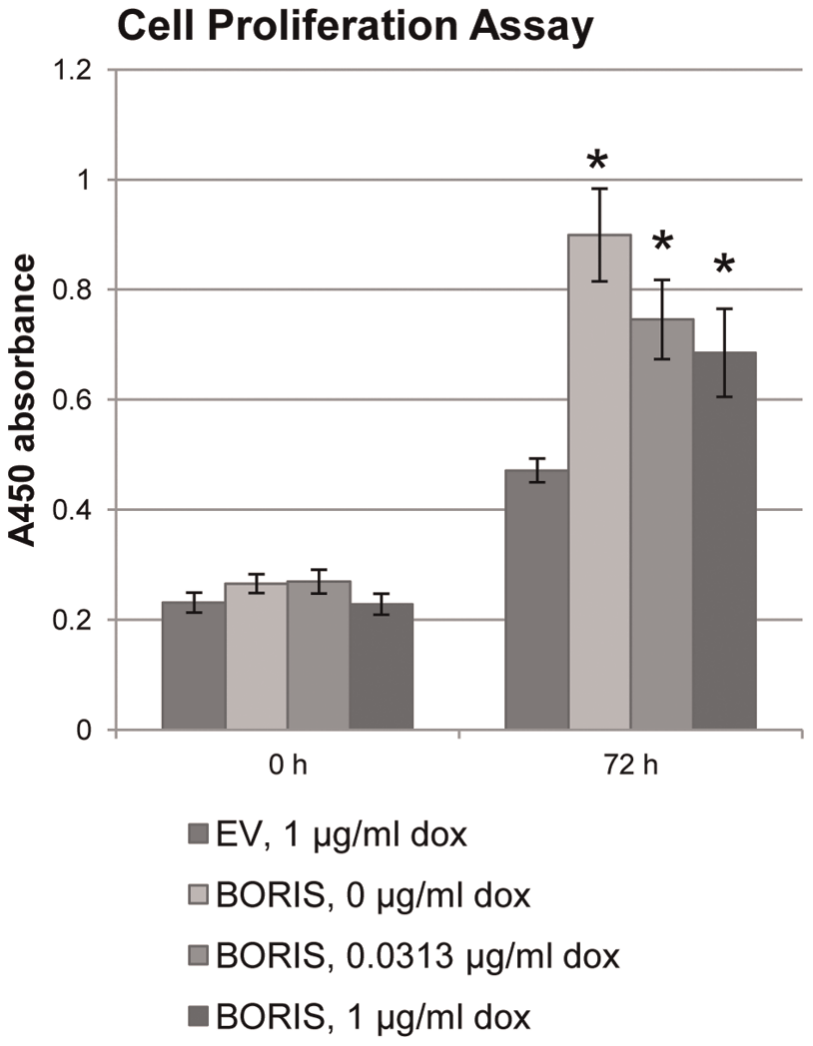
Smaller concentrations of BORIS induce H358 cell growth. Cell proliferation assay for H358 cells transfected with BORIS or control empty vectors and induced by indicated doxycycline concentrations. Values are the mean ± SEM of pentaplicate cultures in 96-well dish. (*, p-value <0.04).

We have previously demonstrated that lower BORIS concentrations induce cell proliferation [10]. We have also demonstrated that SBSN stimulates cell growth in lung cancer cell lines [9]. If the dose-dependent effects of BORIS on the activation of its targets are generalized to cell growth, a similar inverse relationship would be expected between *BORIS* expression and overall cell growth. BORIS is able to increase H358 proliferation by 90% at lower concentrations of BORIS (Figure 6, 0 µg/ml doxycycline). Interestingly, high BORIS concentrations lead to a relative decrease in cell proliferation (Figure 6), in agreement with the effects of BORIS on *SBSN* gene expression. The data were independently confirmed on A549 (Figure S7), HeLa (Figure S8), and normal keratinocyte NOK-SI cell lines (Figure S9). We also found this in mouse embryonic 3T3-NIH cells; BORIS expression in rodents is restricted to primary spermatocytes, as in humans [40]. We performed cell proliferation and colony formation assays to demonstrate that BORIS induces cell growth at doses of doxycycline as low as 0.0625 µg/ml, while BORIS overexpression at 1 µg/ml doxycycline leads to a subsequent decrease in cell growth and colony formation (Figure S10).

This data suggest that increasing BORIS concentration results in the reduction of cell growth proliferation and colony formation, similar to the effects of BORIS on *SBSN* expression.

## Discussion

BORIS is a proto-oncogenic transcription factor that is normally expressed in the testis and has been found to be overexpressed to different extents in multiple tumor types, including NSCLC (Figure 1 and [1], [2], [3], [4], [6], [9], [10]). This variability in *BORIS* expression is tightly regulated by transcription factors CTCF and p53, by DNA methylation, and by chromosomal amplification of the 20q13 locus [2], [24], [25], [26], [41]. We found *BORIS* amplification in 10 of 28 (36%) NSCLC, which strongly correlates with reported rates of 20q13 amplification in lung cancer [26], [41]. In agreement with its oncogenic properties, anti-BORIS vaccination strategies demonstrate an inhibition in tumor growth in breast cancer mouse models [28], [42].

Even though BORIS seems like a plausible therapeutic target [28], [42], the relative contribution of BORIS to the expression of downstream targets involved in tumorigenesis has been challenging to dissect [21], [22], [23]. While BORIS was implicated in the activation of many genes including CTAs, the presence of BORIS is not always necessary for their expression [3], [6], [7], [21], [22]. In contrast, BORIS was shown to induce both DNA methylation and demethylation of various target genes [3], [20].

We have described in detail at least two distinctive mechanisms by which BORIS concentrations affect expression of *SBSN*, a novel non-CTA target gene. At lower concentrations, BORIS transiently binds to its recognition site next to the CpG island within the *SBSN* gene. This binding leads to a significant decrease in DNA methylation of *SBSN*. The presence of BORIS and semimethylated DNA leads to the effective disruption of chromatin and enrichment of active histone modifications of remaining nucleosomes around the *SBSN* TSS. Overall, these changes lead to the induction of *SBSN* expression, consistent with BORIS's stimulatory effect on target gene expression, and to an increase in cell growth [3], [7]. While both *BORIS* and *SBSN* have cell growth stimulating properties [9], [10], the molecular mechanisms of cell proliferation under *SBSN*- and *BORIS*-stimulated conditions need to be further investigated.

The second mechanism of BORIS dose-dependent gene regulation was shown in experiments with higher BORIS concentrations induced by 1 µg/ml doxycycline. In these experiments, overexpression of BORIS caused only minor changes in DNA demethylation and chromatin reorganization, as compared to controls (Table 1). Thus, an increase in BORIS concentration, caused by increasing doxycycline doses to 1 µg/ml, induced a relative increase in DNA methylation of *SBSN*. This correlates with the observed relative enrichment in repressive chromatin modifications and partial chromatin condensation (Table 1). These conditions lead to moderate induction of *SBSN* gene expression by BORIS, where cell proliferation was either only slightly increased, unaffected, or even decreased.

**Table 1:** Dose-dependent changes in *SBSN* parameters induced by BORIS.

	EV,1 µg /ml dox	BORIS,0 µg/ml dox	BORIS,0.03 µg/ml dox	BORIS,1 µg/ml dox
*BORIS* expression	**−**	**+**	**++**	**+++**
BORIS occupancy	**−**	**+**	**++**	**+++**
*SBSN* expression	**−**	**+++**	**++**	**+**
*SBSN* methylation	**++++**	**+**	**++**	**+++**
H3K4me3 enrichment	**−**	**+++**	**+**	**−**
H3Ac enrichment	**−**	**+++**	**++**	**−**
H3K9me3 enrichment	**+**	**−**	**−**	**+**
Nucleosome occupancy	**+++**	**−**	**++**	**+**
Cell growth	**−**	**+++**	**++**	**+**

Relative changes in different parameters were ranked from (−) to (++++) in according to results from Figures 1, 2, 3, 4, and 5.

We have also demonstrated that *SBSN* is a direct target of BORIS epigenetic regulation, where BORIS binds close to the CpG island of *SBSN* to directly regulate the methylation status of its target. This statement is supported by data demonstrating that all BORIS concentrations induce *SBSN* expression, while knock-down of *BORIS* leads to strong decrease in *SBSN* expression (Figure S4). We speculate that downregulation of *SBSN* in this case is mediated by DNA methylation and repression of chromatin organization. BORIS-dependent stimulatory effects directly correlate with an overall decrease in methylation of the *SBSN* CpG island, supporting previously published data that BORIS stimulates expression of target genes via DNA demethylation [1], [2], [6], [7], [10]. However, there is an inverse correlation between expression of BORIS and of its target genes; only lower BORIS concentrations induce the highest expression of its target gene, while higher BORIS concentrations lead to a smaller stimulatory effect. Such an inverse relationship was shown for *SBSN* (this work), *H19* and *TKTL* (data not shown) and has been noticed in the scientific literature for *hTERT, RB2/p130* and *TSP50* [4], [13], [43]. This effect correlates with effects on cell growth, where lower BORIS concentrations led to the greatest stimulatory effects on cell growth and higher BORIS concentrations led to slower cell proliferation or even regression in cell growth. We also conclude that the induction of *SBSN* at lower BORIS concentrations and the relative decrease in *SBSN* induction at higher BORIS concentrations is not lung-specific but more likely a universal phenomenon. The lower BORIS concentrations induced in this study are within the physiological range of BORIS concentrations found in clinical samples, while higher BORIS concentrations induced by 1 µg/ml doxycycline are 10 times higher than in clinical samples (compare Figure 1 and Figure S1). Therefore, we do not exclude the possibility that the relative inhibitory effect of BORIS is induced by superphysiological BORIS concentrations at 1 µg/ml doxycycline. Vatolin and colleagues argue that induction of *BORIS* even with 2 µg/ml doxycycline is not toxic to cultured cells [6]. However the indirect toxic effect of *BORIS* needs to be further investigated. The data presented here imply that some of these differential effects of BORIS noted previously in the literature may be attributable to a dose-dependent effect of BORIS on downstream target activation.

We also speculate that BORIS participates in the stimulation of *SBSN* expression via recruitment of additional factors, so that only small concentrations of BORIS are required for maximal effect but further increases in BORIS concentration do not lead to further stimulatory effect. Expression of *SBSN* in the absence of *BORIS* in some clinical samples (Figure 1) also suggests that BORIS is sufficient but not required for *SBSN* expression and that concurrent mechanisms of *SBSN* activation occur [12], [21].

Published data suggest the importance of both BORIS and CTCF factors for regulation of expression of target genes in cancerous cells [4], [16], [19], [43]. The highly similar DNA-binding 11 zinc finger domains of BORIS and CTCF [1] and the established interaction of the CTCF C-terminus with its 11 zinc finger domain resulting in dimer formation [44] suggest the cooperative binding of these two factors to the same DNA region, as well as possible co-recruitment to these sites [3], [4], [44]. While expression of CTCF and BORIS are mutually exclusive in normal cells, high mRNA levels of both CTCF and BORIS were detected in different cancerous cells [2], [3], [4]. The presence of both CTCF and BORIS at *hTERT* and *RBL2/p130* promoters has been previously demonstrated [4], [43]. Knock-down of either CTCF or BORIS in an ovarian cell line led to a decrease in expression of the target gene *hTERT* (Figure 5B at [4]), suggesting the importance of the presence of both factors for the regulation of target gene expression.

To evaluate the possibility that CTCF also plays a role in the regulation of *SBSN* expression, we analyzed CTCF binding with *SBSN* DNA. ChIP analysis revealed preferential CTCF binding to downstream CTCF/BORIS binding sites at the *SBSN* gene in a manner similar to the control *NY-ESO-1* gene (Figure S11). We also observed the unexpected transient 2-fold enrichment of CTCF binding at lower BORIS concentrations (0 µg/ml doxycycline) compared to control. A gradual increase in BORIS concentrations displaces CTCF from CTCF/BORIS binding sites at both *SBSN* and *NY-ESO-1* genes (Figure S11) without significant effect on *CTCF* expression (Figure S12; [7]).

In agreement with published studies, we found that both CTCF displacement by BORIS at 1 µg/ml doxycycline and BORIS knock-down led to a decrease in *SBSN* gene expression (Figures 1, S4 and S11). These data imply that the presence of both transcription factors CTCF and BORIS is important for maximal cancer-specific *SBSN* activation. We observed that displacement of CTCF correlates with decreased *SBSN* expression, suggesting that CTCF can participate in the stimulation of *SBSN* expression via recruitment of additional factors of transcription machinery, according to prior reports [19], [45], [46].

Intriguingly, BORIS binding to *SBSN* was increased only by 20% at 0 µg/ml doxycycline concentration according to quantitative real time PCR (qRT-PCR) from BORIS-ChIP DNA (Figure 2C), whereas BORIS expression was induced by 1,500 times. This can be explained by strong DNA demethylation and increased CTCF binding to less methylated DNA [24], [47]. Although BORIS binding to the negative control *c-MYC-G* site was very low, we observed a minor increase in occupancy with increased BORIS concentration (Figure 2C), which may be affected by signal from distal BORIS/CTCF binding sites at *c-MYC* [24].

We also noticed prominent CTCF displacement from BORIS/CTCF sites at the *SBSN* gene (Figure 2C and S11). CTCF displacement by BORIS mimics the physiological condition of mutually exclusive expression of *CTCF* and *BORIS*. In addition, the *BORIS*-bearing 20q13 locus is often amplified during carcinogenesis, while the *CTCF*-bearing 16q22 locus undergoes loss of heterozygosity (LOH) [2]. Such co-existing genetic events affect the same pathway and significantly deregulates BORIS/CTCF-controlled genes [2]. BORIS has multiple splice variants, which may each have different effects on expression of *SBSN* and of other BORIS isoforms. We do not exclude the possibility that overexpression of the largest BORIS isoform (B0 isoform; [48]) used in this work may affect expression of other BORIS variants via complex positive- and negative-feedback loops. The balance between different BORIS isoforms is likely important for the regulation of BORIS target genes such as *SBSN*. Investigation of the effects of these individual BORIS isoforms may also yield insight into the varied effects of BORIS on downstream effectors.

## Supporting Information

Figure S1 ***BORIS*** **and** ***SBSN*** **expression in H358.** GAPDH-normalized *BORIS* and *SBSN* expression induced by indicated concentrations of doxycycline (dox). Expression was quantified relative to GAPDH. The Y scale is the same as in Figure 1. (*, p-value <0.00006 (*t* test)).
(TIF)

Figure S2 **BORIS-dependent activation of** ***SBSN*** **gene expression.** Relative *BORIS* (A, C) and *SBSN* (B, D) mRNA levels in A549 (A, B) and HeLa (C, D) cell lines after transient transfection of BORIS. *BORIS* expression was induced by indicated concentrations of doxycycline (dox) 24 hours after transfection with control empty vector or BORIS expressing vector. Expression was quantified relative to GAPDH with the control (EV) referred as 1 (*, p-value <0.000005 (A), p-value <0.002 (B), p-value <0.00009 (C), p-value <0.004 for (D); unlabeled bars are p-value >0.05).
(TIF)

Figure S3 **Relative expression of** ***SBSN*** **and** ***BORIS*** **in lung cancer cell lines.** Relative *BORIS* (A) and *SBSN* (B) expression in H358 and H1299 cell lines. P-values are indicated.
(TIF)

Figure S4 **BORIS is required for** ***SBSN*** **expression in H1299 cell line.** Relative *BORIS* (A) and *SBSN* (B) mRNA levels after knock–down of BORIS expression in the H1299 cell line. BORIS specific shRNA was used for knocking down BORIS expression as described in Materials and Methods S1. Expression was quantified by qRT-PCR 48 hours after transfection with anti-BORIS shRNA or control scrambled shRNA. Expression was quantified relative to GAPDH with the control referred as 1 (*, p-value <0.05 for (A); p-value <0.00008 for (B)).
(TIF)

Figure S5 ***NY-ESO-1*** **chromatin structure reorganization upon BORIS induction.** Chromatin immunoprecipitation assay with antibodies to histone modifications – H3K4me3 (A), H3Ac (B) and H3K9me3 (C). Enrichment of specific histone modifications near *NY-ESO-1* transcription start site (TSS) was measured for H358 cells transfected with BORIS and induced by indicated doxycycline concentrations. Experiment was performed as described in (Fig. 3). *, p-value <0.02 (A), p-value <0.04 (B), p-value <0.05 (C).
(TIF)

Figure S6 **Chromatin isolation from H358 after BORIS induction.** Time course digestion of H358 cell line nuclei after BORIS induction with micrococcal nuclease (MNase). *BORIS* expression was induced by 0, 0.0313 or 1 µg/ml doxycycline 24 hours after transfection with control empty vector or with BORIS expressing vector. Cell nuclei were isolated and digested with MNase for various time intervals (2, 4, 8, 30, or 60 min), and the DNA was purified and analyzed on a 1% agarose gel. A representative picture for BORIS transfection at 0 µg/ml doxycycline is shown.
(TIF)

Figure S7 **Effect of different BORIS concentrations on A549 cell proliferation.** Cells were transfected with BORIS or control empty vectors and induced by indicated doxycycline concentrations. Cell count was calculated 48 hours after doxycycline induction as described in the Methods. Values are the mean ± SEM of pentaplicate cultures in 96-well dish. (*, p-value <0.003).
(TIF)

Figure S8 **Effect of different BORIS concentrations on HeLa cell proliferation.** Cells were transfected with BORIS or control empty vectors and induced by indicated doxycycline concentrations. Cell count was calculated 48 hours after doxycycline induction as described in the Methods. Values are the mean ± SEM of pentaplicate cultures in 96-well dish. (*, p-value <0.003; unlabeled bars are p-value >0.05).
(TIF)

Figure S9 **Effect of different BORIS concentrations on normal keratinocyte NOK-SI cell proliferation.** (A) Relative *BORIS* mRNA level in NOK-SI cell line after BORIS transient transfection. *BORIS* expression was induced by indicated concentrations of doxycycline (dox) 24 hours after transfection with control empty vector or BORIS expressing vector. Expression was quantified relative to GAPDH with the control (EV) referred as 1 (*, p-value <0.00003). (B) Cell proliferation after transient transfection of BORIS. Cell count was calculated 72 hours after doxycycline induction as described in the Methods. Values are the mean ± SEM of pentaplicate cultures in 96-well dish. (*, p-value <0.05).
(TIF)

Figure S10 **Effect of different BORIS concentrations on mouse embryonic 3T3-NIH cell proliferation.** Stable clones of 3T3-NIH with genome-intercalated empty vector or BORIS sequences were used after BORIS induction with indicated doxycycline concentrations. (A) Relative *BORIS* mRNA level in 3T3-NIH clone, quantified relative to GAPDH with the control (EV) referred as 1 (*, v-value <0.002). (B) Cell proliferation after 0 and 72 hours doxycycline induction (*, v-value <0.003). (C) Formation of colonies by 3T3-NIH 2 weeks after BORIS induction by indicated doxycycline concentrations, performed as described in Materials and Methods S1 (*, v-value <0.00004). (D) Capture of colonies formed in the experiment from (C).
(TIF)

Figure S11 **CTCF is recruited to** ***SBSN*** **upon BORIS induction.** CTCF enrichment at CTCF/BORIS binding sites at *SBSN*, at positive control *NY-ESO-1*, or at negative control *hMYC-G*, as analyzed by qRT-PCR from ChIP DNA. Note that BORIS and CTCF bind the same DNA motifs (shown in Fig. 1A). The lysate from (Fig. 1C) was used for ChIP experiments with CTCF-specific antibody. Values are normalized to non-specific rabbit IgG (*, p-value <0.04; unlabeled bar, p-value >0.05).
(TIF)

Figure S12 **CTCF expression does not depend on BORIS concentration.** Relative *CTCF* mRNA levels in the H358 cell line after transient transfection with BORIS. *BORIS* expression was induced by indicated concentrations of doxycycline (dox) 24 hours after transfection with control empty vector or BORIS expressing vector. Expression was quantified 48 hours after doxycycline induction relative to GAPDH with the control (EV) referred as 1. No significant changes in CTCF expression were identified.
(TIF)

Table S1 **Sequences of the primers and probes.**
(XLS)

Materials and Methods S1 (DOC)
