# Supplementary figures and images for: Dose-Dependent Activation of Putative Oncogene SBSN by BORIS
Source: PLoS One. 2012 Jul 5;7(7):e40389. doi: 10.1371/journal.pone.0040389 (PMC3390376; doi:10.1371/journal.pone.0040389)

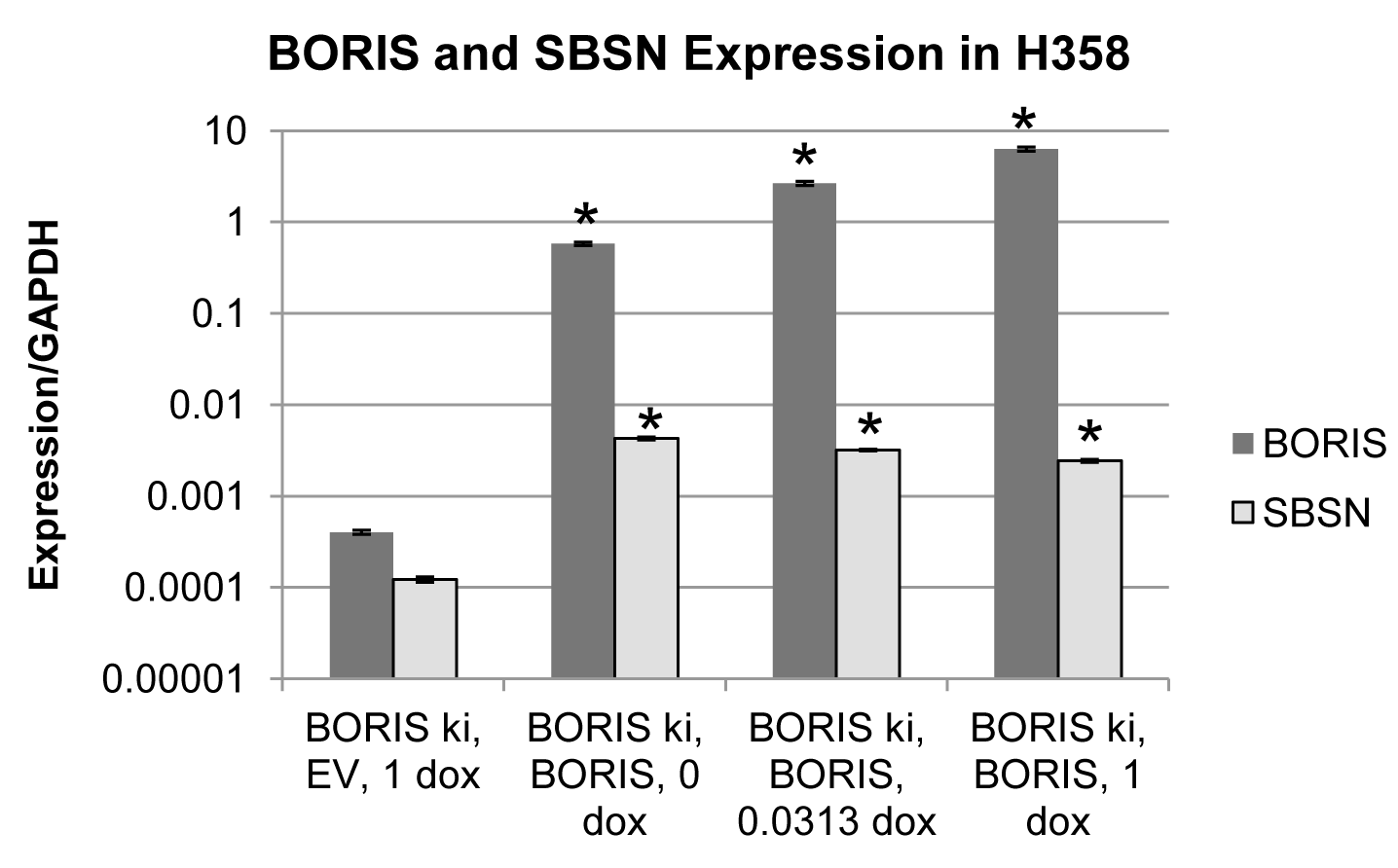

Supplement: Figure S1 — BORIS and SBSN expression in H358. GAPDH-normalized BORIS and SBSN expression induced by indicated concentrations of doxycycline (dox). Expression was quantified relative to GAPDH. The Y scale is the same as in Figure 1. (*, p-value <0.00006 (t test)). (TIF) [file pone.0040389.s001.tif]

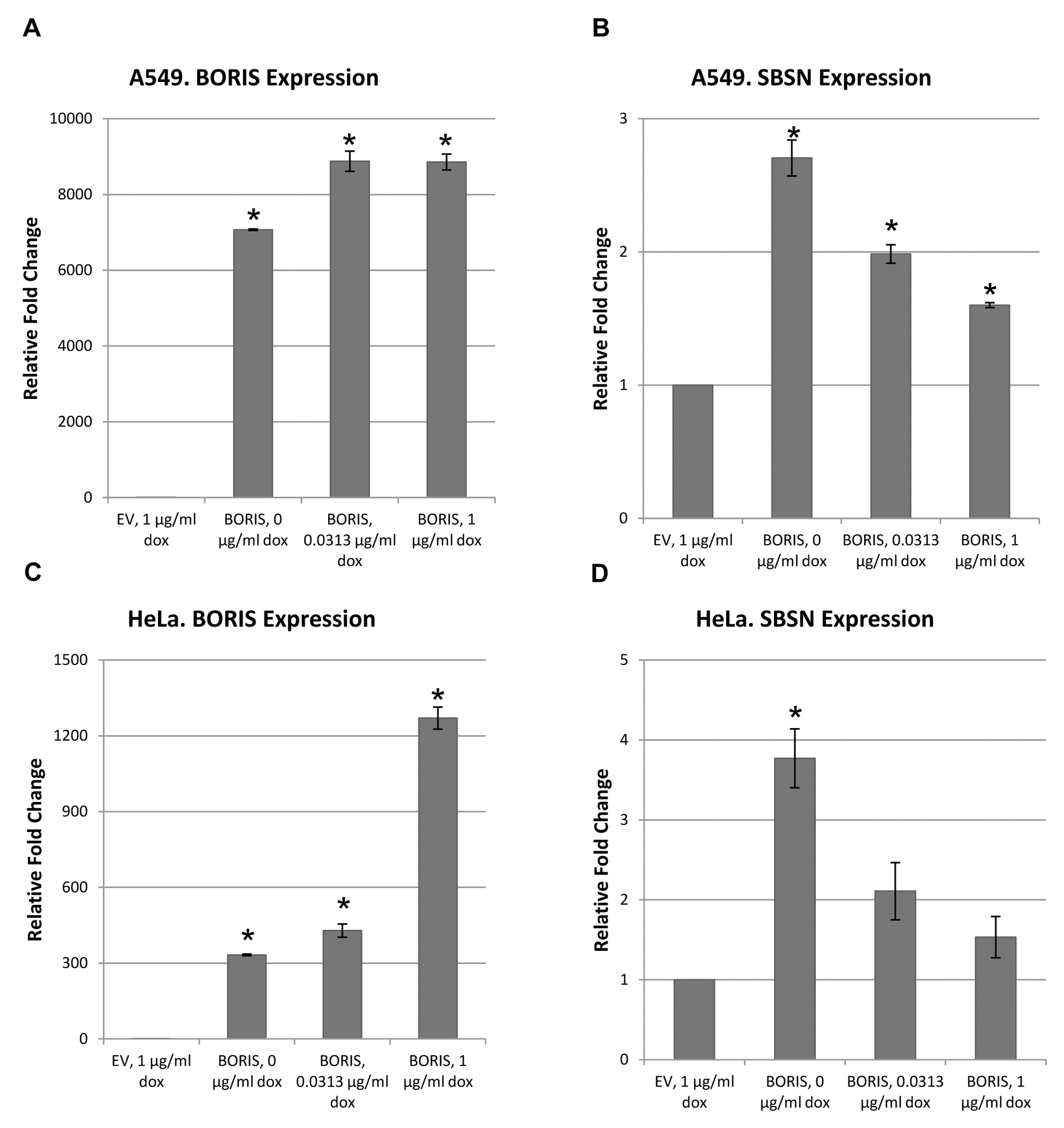

Supplement: Figure S2 — BORIS-dependent activation of SBSN gene expression. Relative BORIS (A, C) and SBSN (B, D) mRNA levels in A549 (A, B) and HeLa (C, D) cell lines after transient transfection of BORIS. BORIS expression was induced by indicated concentrations of doxycycline (dox) 24 hours after transfection with control empty vector or BORIS expressing vector. Expression was quantified relative to GAPDH with the control (EV) referred as 1 (*, p-value <0.000005 (A), p-value <0.002 (B), p-value <0.00009 (C), p-value <0.004 for (D); unlabeled bars are p-value >0.05). (TIF) [file pone.0040389.s002.tif]

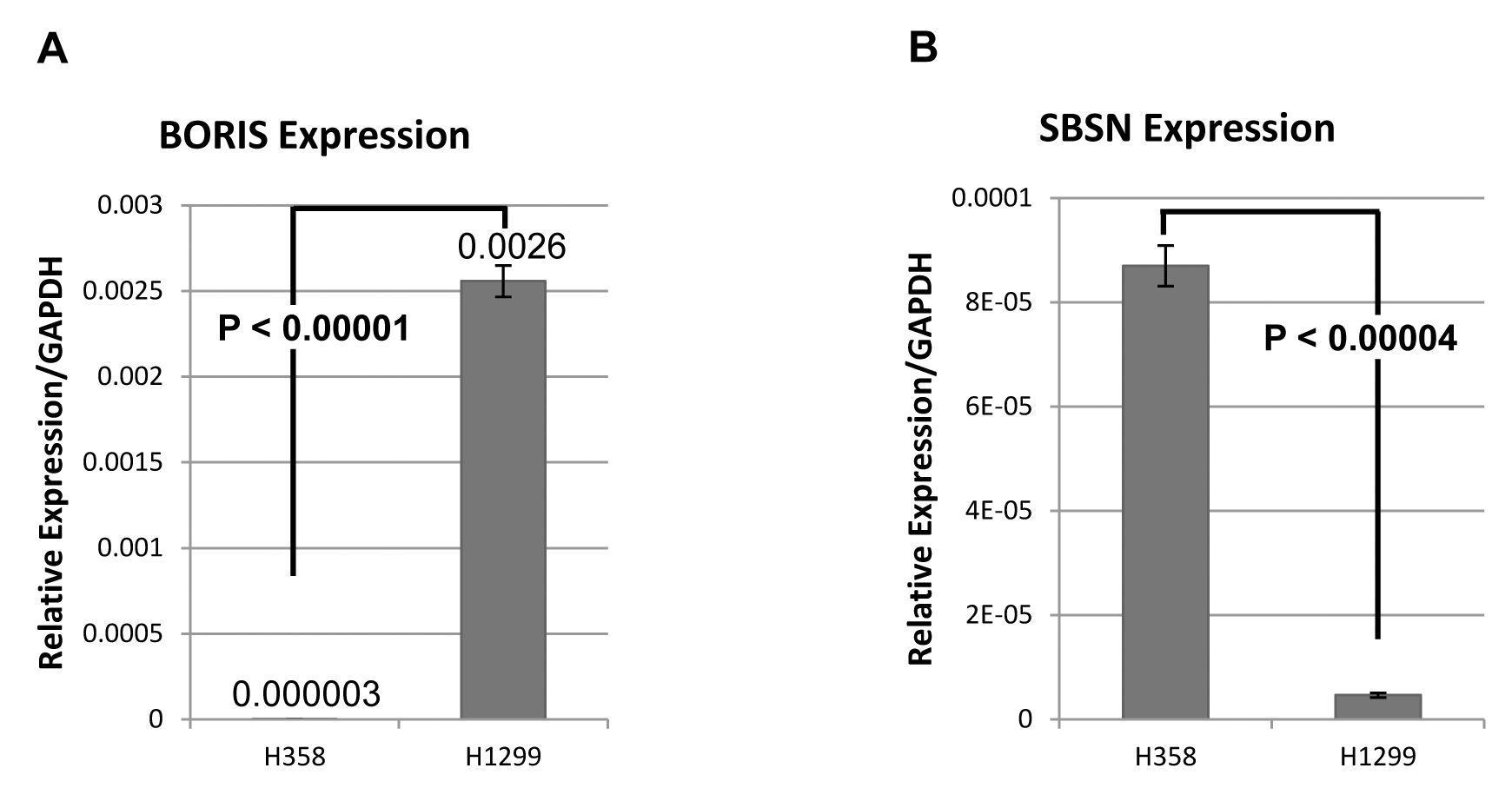

Supplement: Figure S3 — Relative expression of SBSN and BORIS in lung cancer cell lines. Relative BORIS (A) and SBSN (B) expression in H358 and H1299 cell lines. P-values are indicated. (TIF) [file pone.0040389.s003.tif]

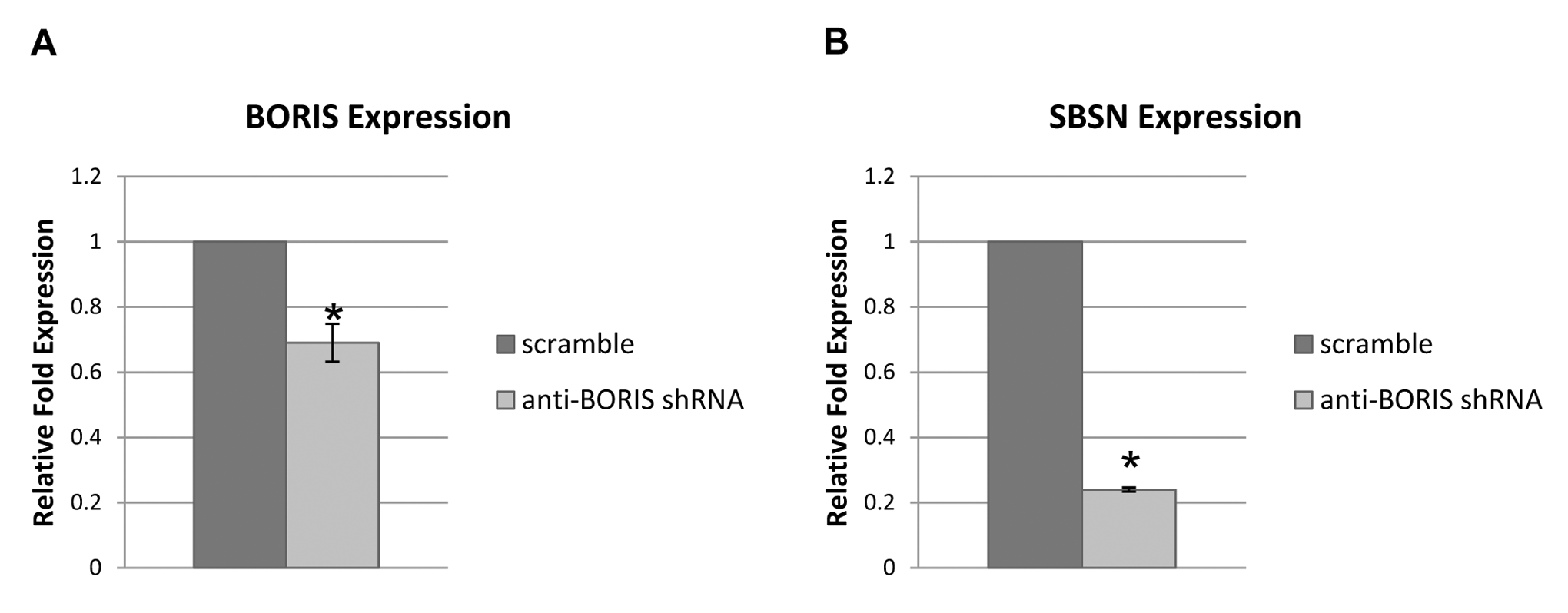

Supplement: Figure S4 — BORIS is required for SBSN expression in H1299 cell line. Relative BORIS (A) and SBSN (B) mRNA levels after knock–down of BORIS expression in the H1299 cell line. BORIS specific shRNA was used for knocking down BORIS expression as described in Materials and Methods S1. Expression was quantified by qRT-PCR 48 hours after transfection with anti-BORIS shRNA or control scrambled shRNA. Expression was quantified relative to GAPDH with the control referred as 1 (*, p-value <0.05 for (A); p-value <0.00008 for (B)). (TIF) [file pone.0040389.s004.tif]

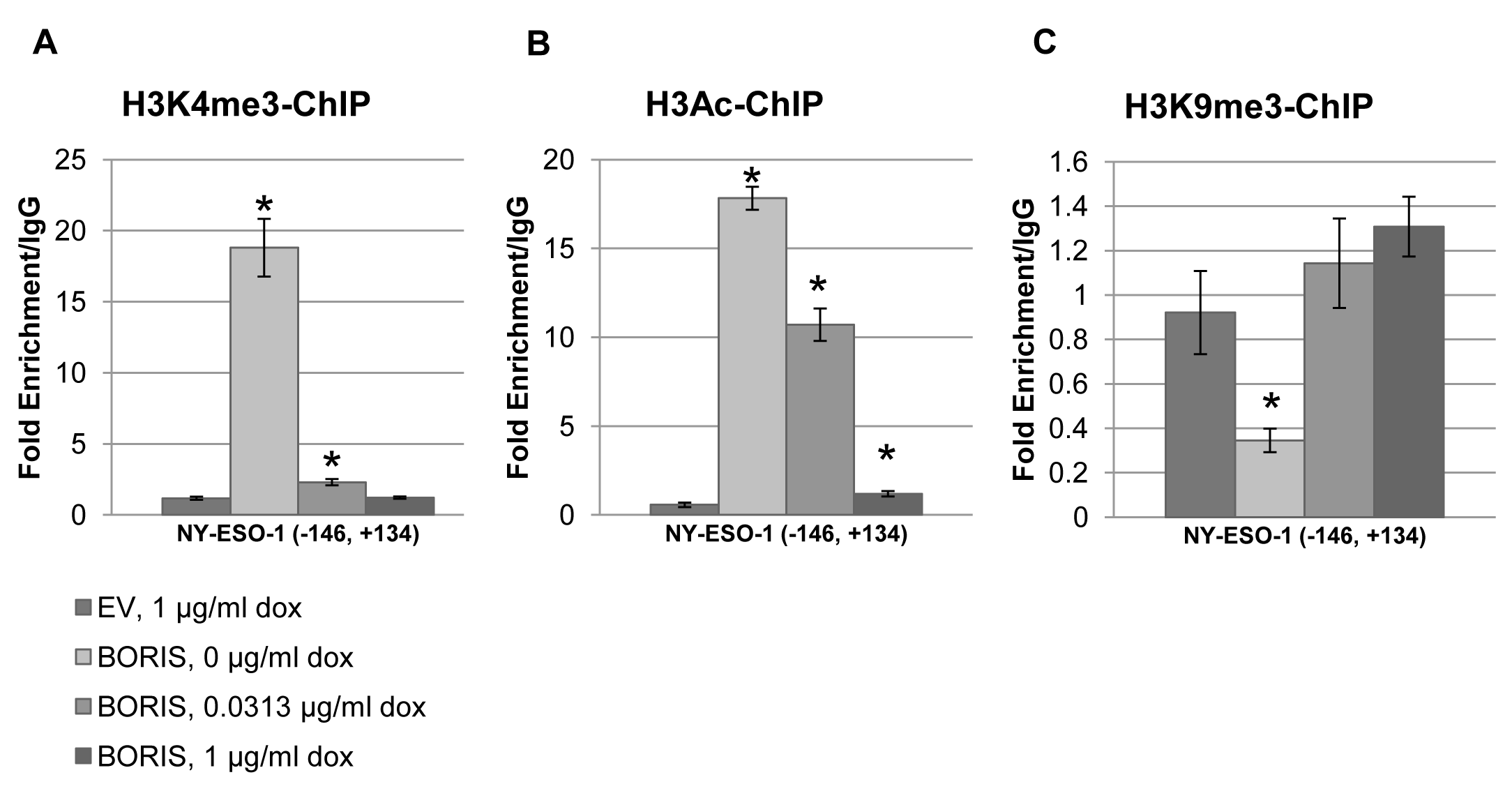

Supplement: Figure S5 — NY-ESO-1 chromatin structure reorganization upon BORIS induction. Chromatin immunoprecipitation assay with antibodies to histone modifications – H3K4me3 (A), H3Ac (B) and H3K9me3 (C). Enrichment of specific histone modifications near NY-ESO-1 transcription start site (TSS) was measured for H358 cells transfected with BORIS and induced by indicated doxycycline concentrations. Experiment was performed as described in (Fig. 3). *, p-value <0.02 (A), p-value <0.04 (B), p-value <0.05 (C). (TIF) [file pone.0040389.s005.tif]

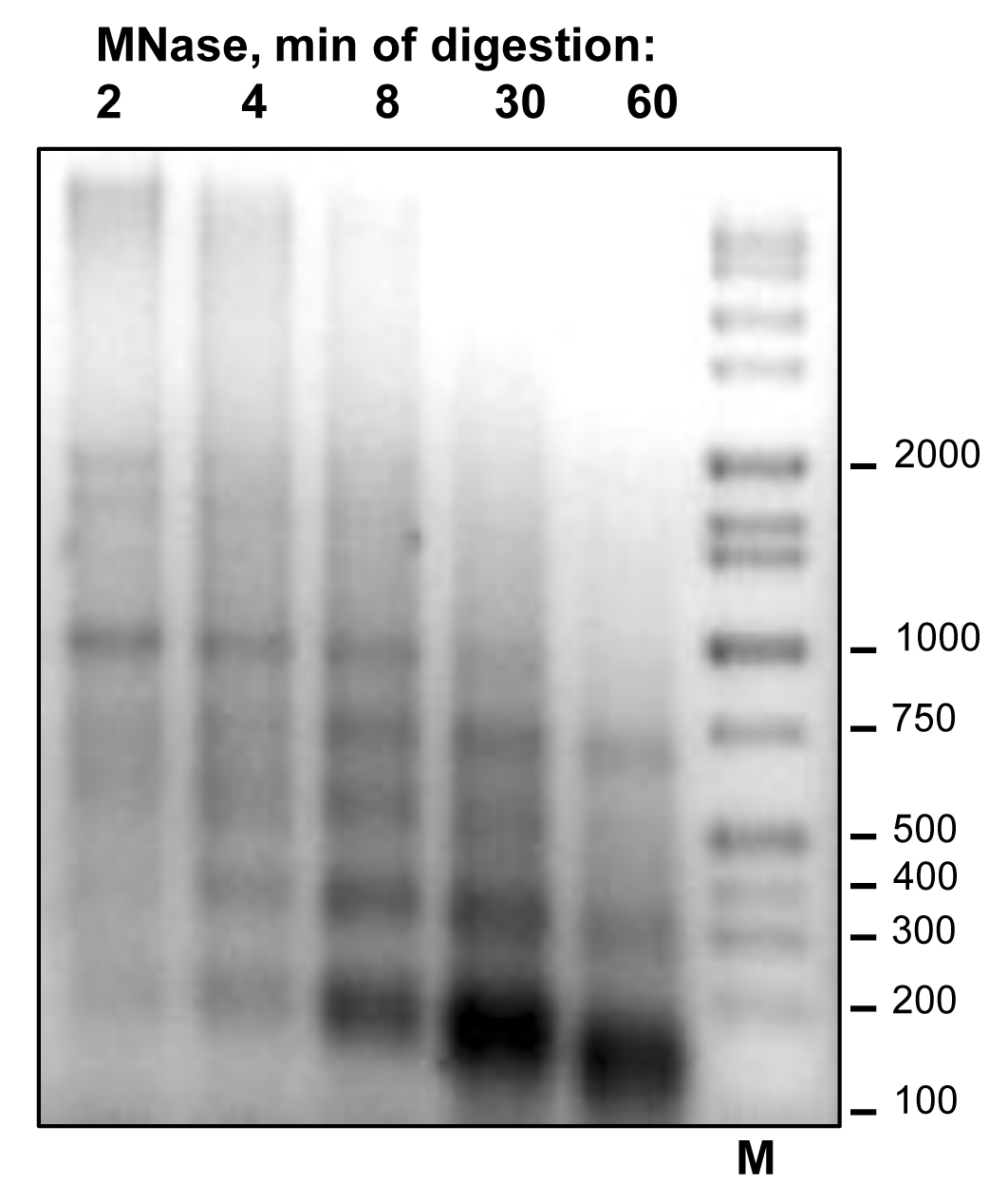

Supplement: Figure S6 — Chromatin isolation from H358 after BORIS induction. Time course digestion of H358 cell line nuclei after BORIS induction with micrococcal nuclease (MNase). BORIS expression was induced by 0, 0.0313 or 1 µg/ml doxycycline 24 hours after transfection with control empty vector or with BORIS expressing vector. Cell nuclei were isolated and digested with MNase for various time intervals (2, 4, 8, 30, or 60 min), and the DNA was purified and analyzed on a 1% agarose gel. A representative picture for BORIS transfection at 0 µg/ml doxycycline is shown. (TIF) [file pone.0040389.s006.tif]

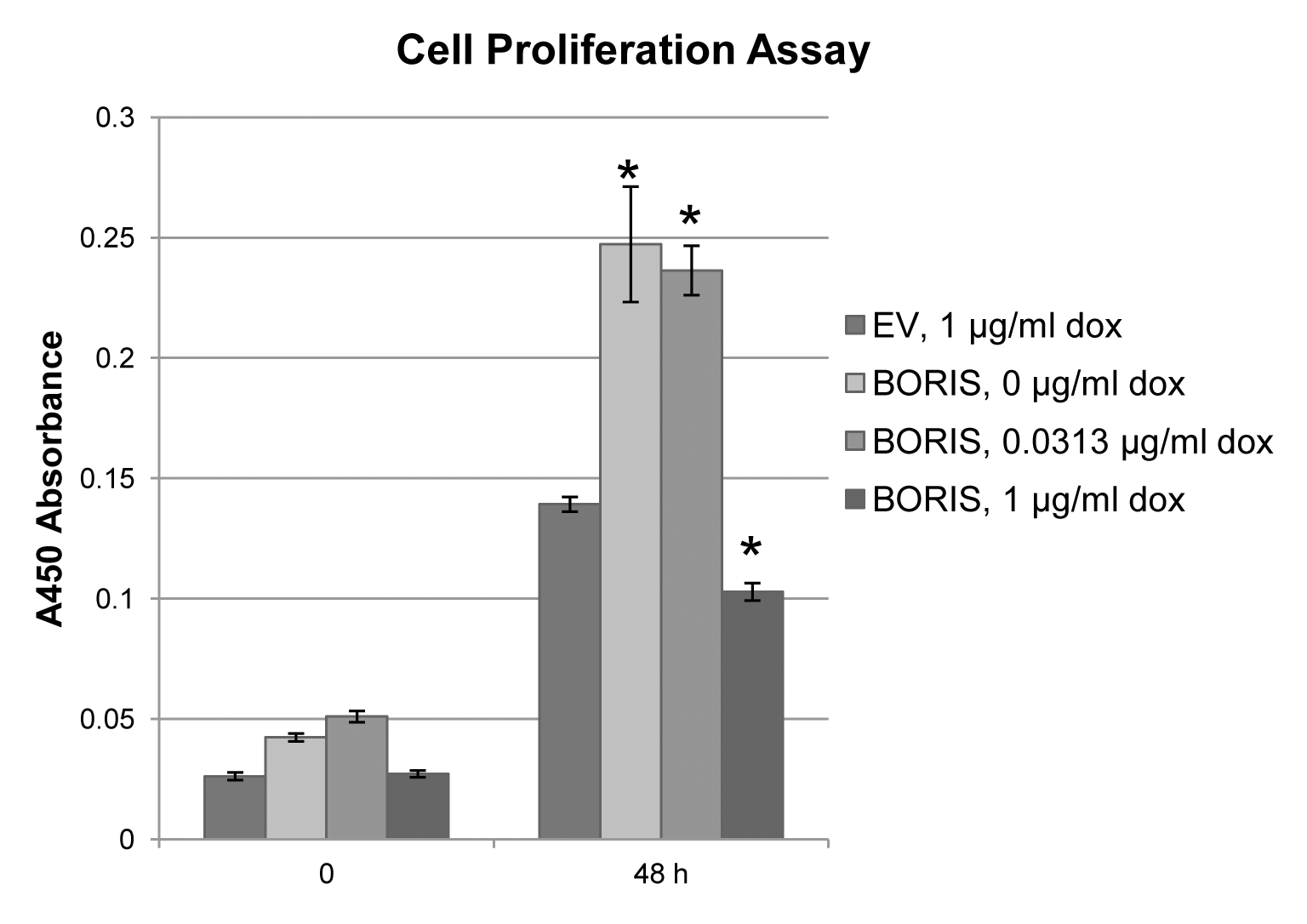

Supplement: Figure S7 — Effect of different BORIS concentrations on A549 cell proliferation. Cells were transfected with BORIS or control empty vectors and induced by indicated doxycycline concentrations. Cell count was calculated 48 hours after doxycycline induction as described in the Methods. Values are the mean ± SEM of pentaplicate cultures in 96-well dish. (*, p-value <0.003). (TIF) [file pone.0040389.s007.tif]

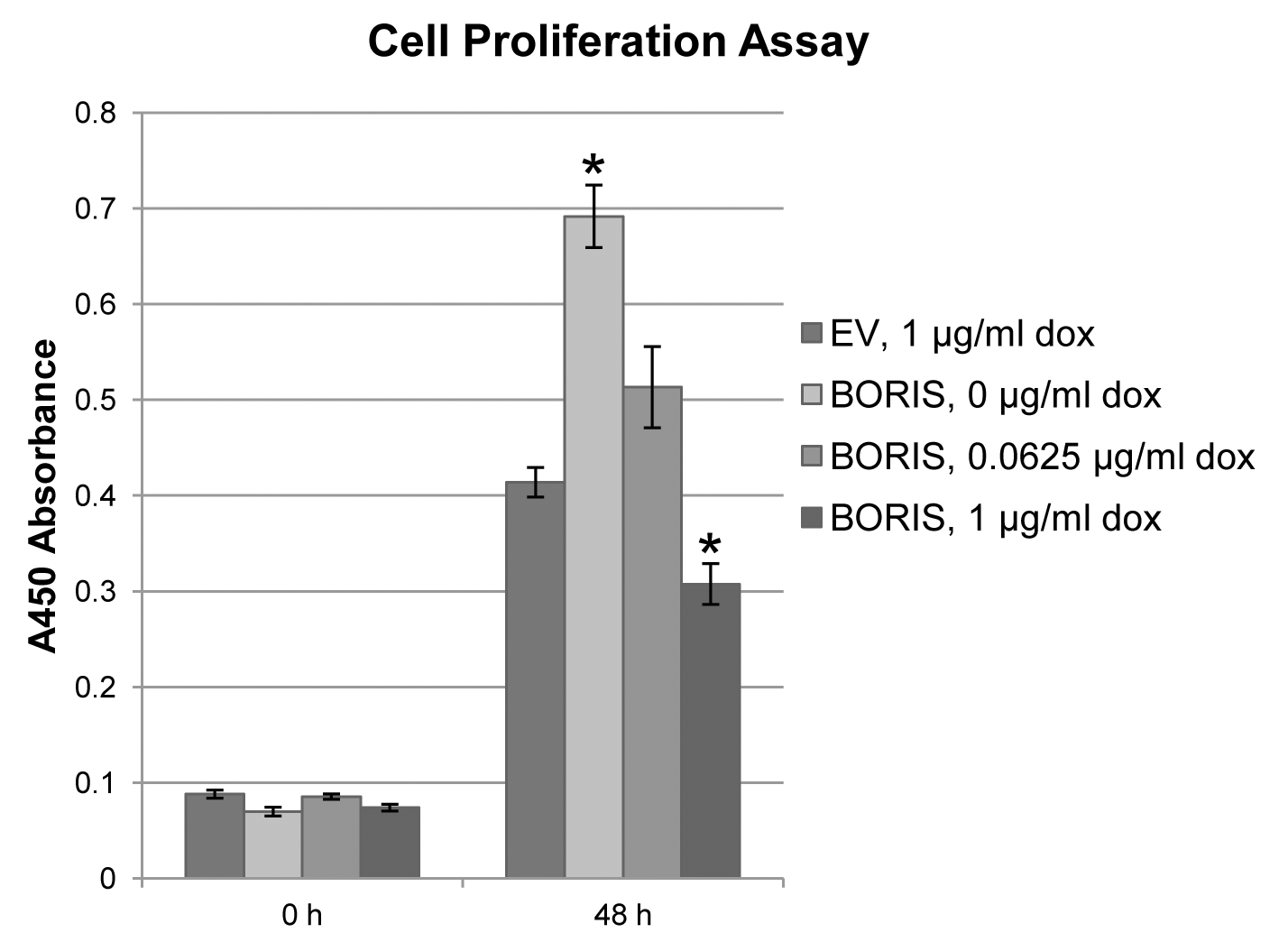

Supplement: Figure S8 — Effect of different BORIS concentrations on HeLa cell proliferation. Cells were transfected with BORIS or control empty vectors and induced by indicated doxycycline concentrations. Cell count was calculated 48 hours after doxycycline induction as described in the Methods. Values are the mean ± SEM of pentaplicate cultures in 96-well dish. (*, p-value <0.003; unlabeled bars are p-value >0.05). (TIF) [file pone.0040389.s008.tif]

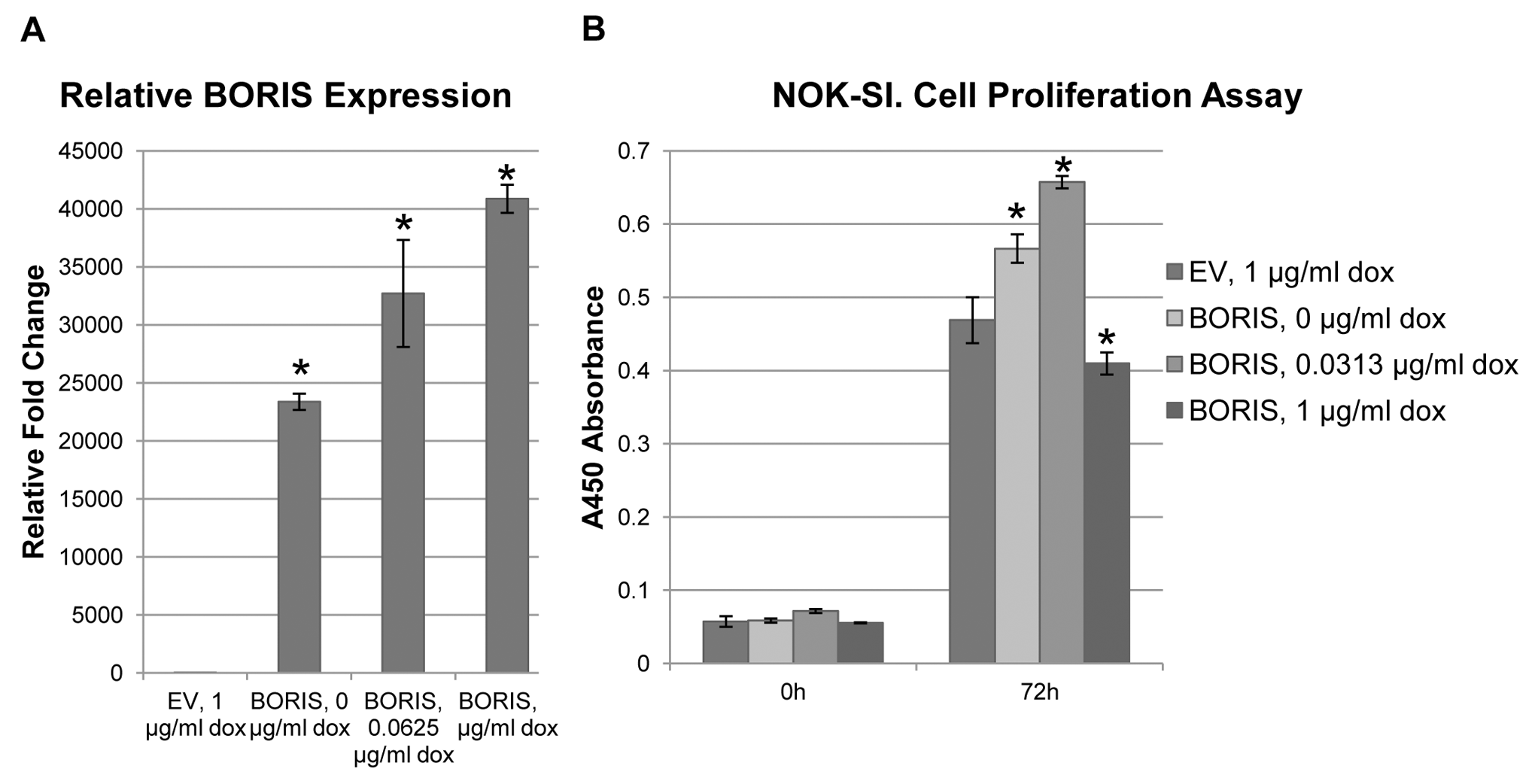

Supplement: Figure S9 — Effect of different BORIS concentrations on normal keratinocyte NOK-SI cell proliferation. (A) Relative BORIS mRNA level in NOK-SI cell line after BORIS transient transfection. BORIS expression was induced by indicated concentrations of doxycycline (dox) 24 hours after transfection with control empty vector or BORIS expressing vector. Expression was quantified relative to GAPDH with the control (EV) referred as 1 (*, p-value <0.00003). (B) Cell proliferation after transient transfection of BORIS. Cell count was calculated 72 hours after doxycycline induction as described in the Methods. Values are the mean ± SEM of pentaplicate cultures in 96-well dish. (*, p-value <0.05). (TIF) [file pone.0040389.s009.tif]

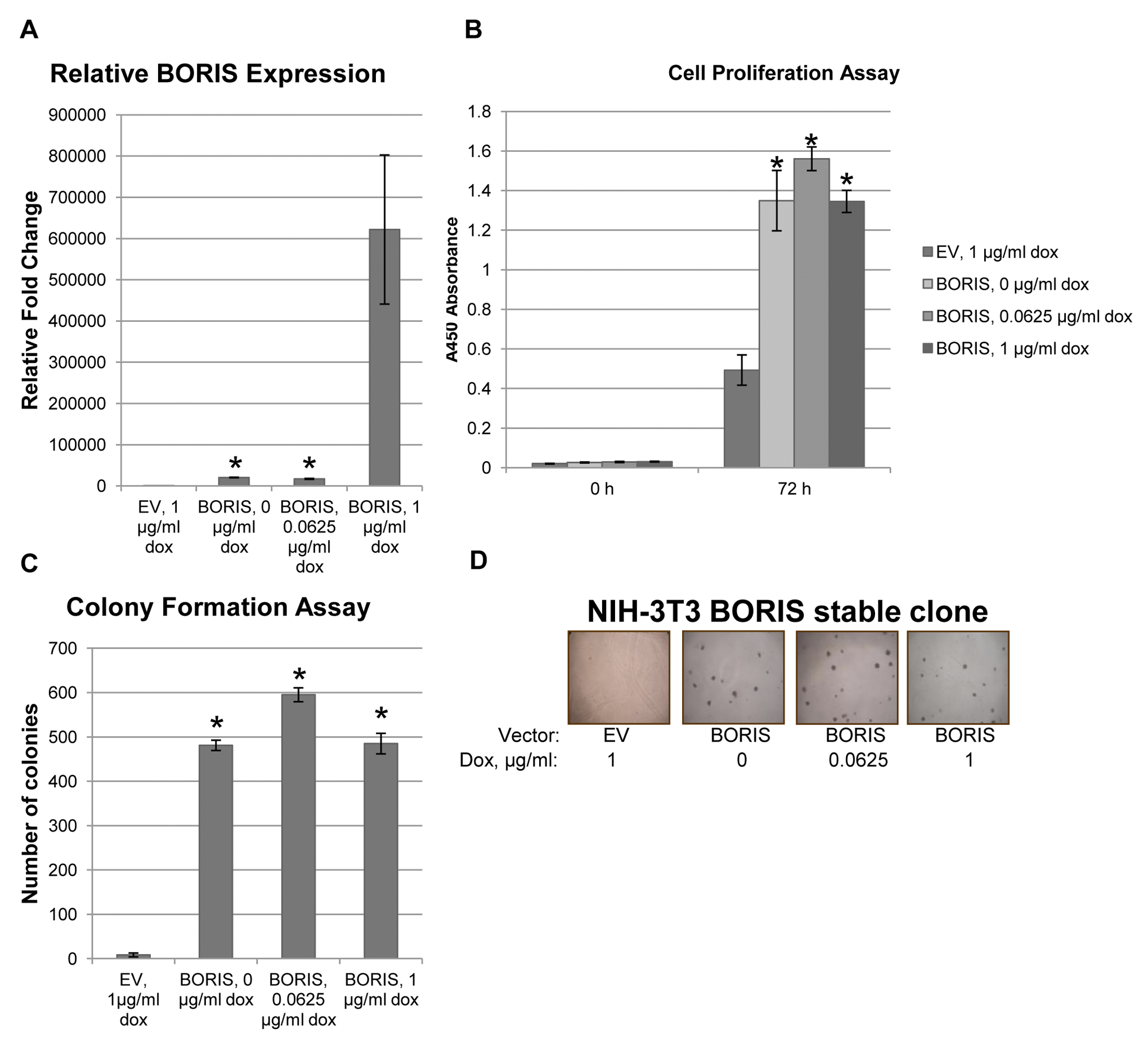

Supplement: Figure S10 — Effect of different BORIS concentrations on mouse embryonic 3T3-NIH cell proliferation. Stable clones of 3T3-NIH with genome-intercalated empty vector or BORIS sequences were used after BORIS induction with indicated doxycycline concentrations. (A) Relative BORIS mRNA level in 3T3-NIH clone, quantified relative to GAPDH with the control (EV) referred as 1 (*, v-value <0.002). (B) Cell proliferation after 0 and 72 hours doxycycline induction (*, v-value <0.003). (C) Formation of colonies by 3T3-NIH 2 weeks after BORIS induction by indicated doxycycline concentrations, performed as described in Materials and Methods S1 (*, v-value <0.00004). (D) Capture of colonies formed in the experiment from (C). (TIF) [file pone.0040389.s010.tif]

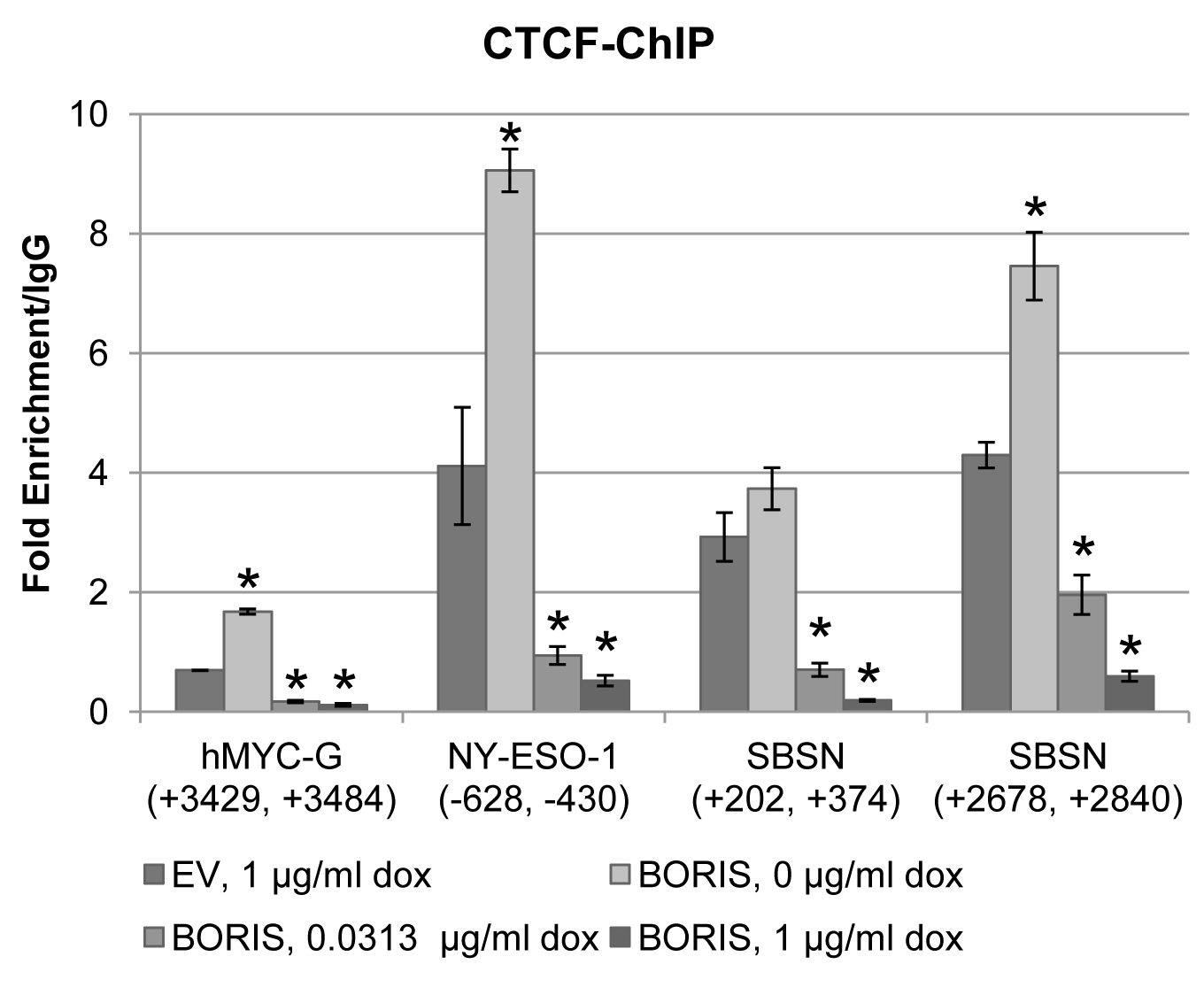

Supplement: Figure S11 — CTCF is recruited to SBSN upon BORIS induction. CTCF enrichment at CTCF/BORIS binding sites at SBSN, at positive control NY-ESO-1, or at negative control hMYC-G, as analyzed by qRT-PCR from ChIP DNA. Note that BORIS and CTCF bind the same DNA motifs (shown in Fig. 1A). The lysate from (Fig. 1C) was used for ChIP experiments with CTCF-specific antibody. Values are normalized to non-specific rabbit IgG (*, p-value <0.04; unlabeled bar, p-value >0.05). (TIF) [file pone.0040389.s011.tif]

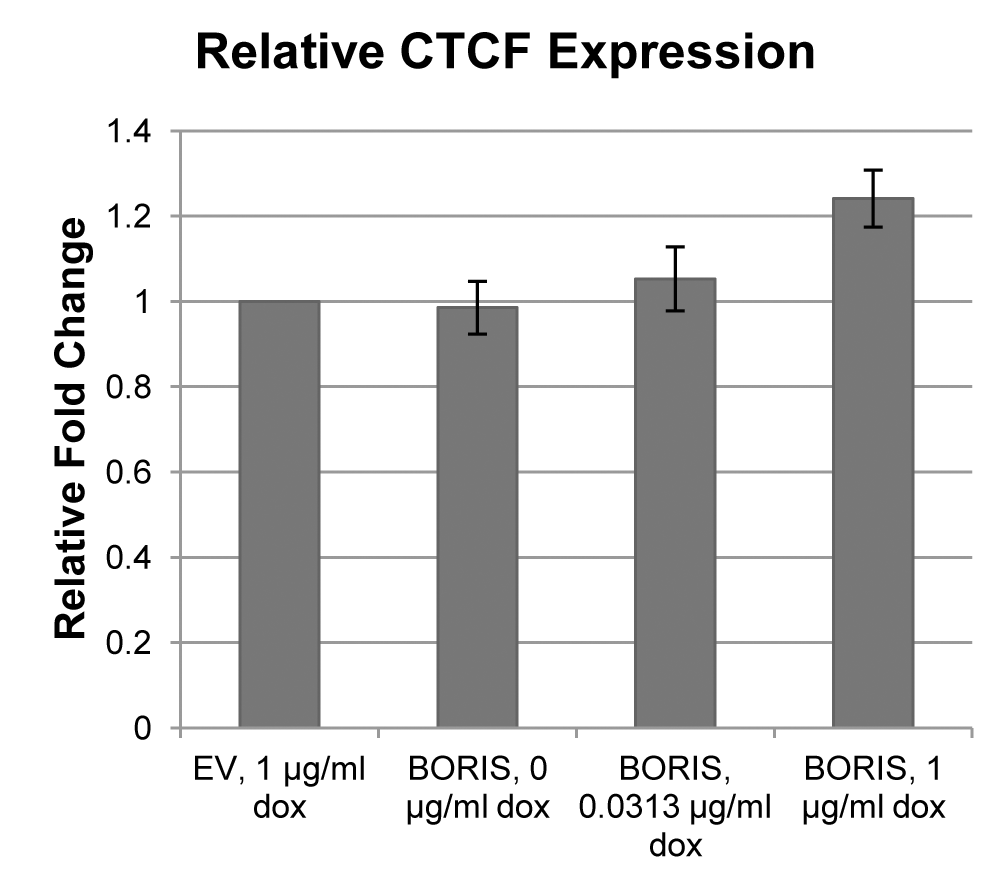

Supplement: Figure S12 — CTCF expression does not depend on BORIS concentration. Relative CTCF mRNA levels in the H358 cell line after transient transfection with BORIS. BORIS expression was induced by indicated concentrations of doxycycline (dox) 24 hours after transfection with control empty vector or BORIS expressing vector. Expression was quantified 48 hours after doxycycline induction relative to GAPDH with the control (EV) referred as 1. No significant changes in CTCF expression were identified. (TIF) [file pone.0040389.s012.tif]
