# Supplementary material for: Dose-Dependent Activation of Putative Oncogene SBSN by BORIS
Source: PLoS One. 2012 Jul 5;7(7):e40389. doi: 10.1371/journal.pone.0040389 (PMC3390376; doi:10.1371/journal.pone.0040389)
Supplement: Materials and Methods S1 — (DOC) [file pone.0040389.s014.doc]

**Dose Dependent Activation of Putative Oncogene *SBSN* by BORIS**

Daria Gaykalova, Rajita Vatapalli, Chad A. Glazer, Sheetal Bhan, Chunbo Shao, David Sidransky, Patrick K. Ha and Joseph A. Califano

**SUPPORTING MATERIALS AND METHODS**

**Cell lines, Plasmids, Transfections and establishment of stable clones**

Lung cancer cell line H1299, lung adenocarcinoma cell line A549, cervix adenocarcinoma cell line HeLa and mouse embryonic 3T3-NIH cell line were obtained from American Type Cell Culture (ATCC). A549, HeLa and 3T3-NIH cell lines were cultured in RPMI1640 medium supplemented with 10% FBS and 1% penicillin/streptomycin. Normal keratinocytes NOK-SI were kindly provided by J. Silvio Gutkind (NIH, Bethesda, MD) and were cultured in “keratinocyte serum-free medium” (Invitrogen, Carlsbad, CA), prepared as directed by manufacturer and supplemented with 1% penicillin/streptomycin. All cells were incubated at 37°C and 5% CO_2_. For ectopic BORIS expression, BORIS expression plasmid (pBIG2i-BORIS, aka BORIS) and control empty vector (EV) were used . A549, HeLa and NOK-SI cells grown in 6-well plates were transfected with 2 µg of the plasmids using Fugene HD (Roche, Indianapolis, IN). Twenty-four hours post-transfection, cells were induced with 0, 0.0313 or 1 µg/ ml doxycycline concentrations in growth media and were allowed to grow for 48 hours before harvesting for RNA or DNA extraction. For establishment of clones with stable expression of empty vector or BORIS, 3T3-NIH cells grown in 100 mm culture dishes were transfected with 15 µg of the plasmids using Fugene HD (Roche) and allowed to grow in presence of 100 μg/ml hygromycin B (Invitrogen). Individual clones were expanded and BORIS expression was evaluated by RT and qPCR after induction of BORIS expression by 0.0625 μg/ml doxycycline. 3T3-NIH stably expressing BORIS grown in 6-well plates were induced by 0, 0.0625 or 1 µg/ ml doxycycline.

**Anchorage-independent growth assay**

Soft agar assays or colony formation assay were conducted after transfection of 3T3-NIH cells with empty vector or BORIS expression vector. Cells were counted and approximately 5000 were added into each 6-well plate. The bottom layer was composed of 0.5% agar, RPMI1640, supplemented with 10% FBS and 1% penicillin/streptomycin, while the cells were suspended in a top layer of 0.35% agar, RPMI1640, supplemented with 10% FBS and 1% penicillin/streptomycin. BORIS expression was induced by 0.0625 μg/ml doxycycline. Cells were incubated at 37°C and 5% CO_2_ for 2 weeks.

**REFERENCES**

1. Basile JR, Castilho RM, Williams VP, Gutkind JS. Semaphorin 4D provides a link between axon guidance processes and tumor-induced angiogenesis. Proc Natl Acad Sci U S A 2006; 103: 9017-22.

2. Bhan S, Negi SS, Shao C, et al. BORIS binding to the promoters of cancer testis antigens, MAGEA2, MAGEA3 and MAGEA4, is associated with their transcriptional activation in lung cancer. Clinical cancer research : an official journal of the American Association for Cancer Research 2011.

3. Vatolin S, Abdullaev Z, Pack SD, et al. Conditional expression of the CTCF-paralogous transcriptional factor BORIS in normal cells results in demethylation and derepression of MAGE-A1 and reactivation of other cancer-testis genes. Cancer Res 2005; 65: 7751-62.
